# Default mode network failure and neurodegeneration across aging and amnestic and dysexecutive Alzheimer’s disease

**DOI:** 10.1093/braincomms/fcad058

**Published:** 2023-03-08

**Authors:** Nick Corriveau-Lecavalier, Jeffrey L Gunter, Michael Kamykowski, Ellen Dicks, Hugo Botha, Walter K Kremers, Jonathan Graff-Radford, Daniela A Wiepert, Christopher G Schwarz, Essa Yacoub, David S Knopman, Bradley F Boeve, Kamil Ugurbil, Ronald C Petersen, Clifford R Jack, Melissa J Terpstra, David T Jones

**Affiliations:** Department of Neurology, Mayo Clinic, Rochester, MN 55905, USA; Department of Radiology, Mayo Clinic, Rochester, MN 55905, USA; Department of Information Technology, Mayo Clinic, Rochester, MN 55905, USA; Department of Neurology, Mayo Clinic, Rochester, MN 55905, USA; Department of Neurology, Mayo Clinic, Rochester, MN 55905, USA; Department of Quantitative Health Sciences, Mayo Clinic, Rochester, MN 55905, USA; Department of Neurology, Mayo Clinic, Rochester, MN 55905, USA; Department of Neurology, Mayo Clinic, Rochester, MN 55905, USA; Department of Radiology, Mayo Clinic, Rochester, MN 55905, USA; Department of Radiology, University of Minnesota, Minneapolis, MN 55455, USA; Department of Neurology, Mayo Clinic, Rochester, MN 55905, USA; Department of Neurology, Mayo Clinic, Rochester, MN 55905, USA; Department of Radiology, University of Minnesota, Minneapolis, MN 55455, USA; Department of Neurology, Mayo Clinic, Rochester, MN 55905, USA; Department of Radiology, Mayo Clinic, Rochester, MN 55905, USA; Department of Radiology, University of Minnesota, Minneapolis, MN 55455, USA; Department of Radiology, University of Missouri, Columbia, MO 65211, USA; Department of Neurology, Mayo Clinic, Rochester, MN 55905, USA; Department of Radiology, Mayo Clinic, Rochester, MN 55905, USA

**Keywords:** dysexecutive Alzheimer’s disease, functional magnetic resonance imaging, network failure quotient, Human Connectome Project-Aging, network biomarkers

## Abstract

From a complex systems perspective, clinical syndromes emerging from neurodegenerative diseases are thought to result from multiscale interactions between aggregates of misfolded proteins and the disequilibrium of large-scale networks coordinating functional operations underpinning cognitive phenomena. Across all syndromic presentations of Alzheimer’s disease, age-related disruption of the default mode network is accelerated by amyloid deposition. Conversely, syndromic variability may reflect selective neurodegeneration of modular networks supporting specific cognitive abilities. In this study, we leveraged the breadth of the Human Connectome Project-Aging cohort of non-demented individuals (*N* = 724) as a normative cohort to assess the robustness of a biomarker of default mode network dysfunction in Alzheimer’s disease, the network failure quotient, across the aging spectrum. We then examined the capacity of the network failure quotient and focal markers of neurodegeneration to discriminate patients with amnestic (*N* = 8) or dysexecutive (*N* = 10) Alzheimer’s disease from the normative cohort at the patient level, as well as between Alzheimer’s disease phenotypes. Importantly, all participants and patients were scanned using the Human Connectome Project-Aging protocol, allowing for the acquisition of high-resolution structural imaging and longer resting-state connectivity acquisition time. Using a regression framework, we found that the network failure quotient related to age, global and focal cortical thickness, hippocampal volume, and cognition in the normative Human Connectome Project-Aging cohort, replicating previous results from the Mayo Clinic Study of Aging that used a different scanning protocol. Then, we used quantile curves and group-wise comparisons to show that the network failure quotient commonly distinguished both dysexecutive and amnestic Alzheimer’s disease patients from the normative cohort. In contrast, focal neurodegeneration markers were more phenotype-specific, where the neurodegeneration of parieto-frontal areas associated with dysexecutive Alzheimer’s disease, while the neurodegeneration of hippocampal and temporal areas associated with amnestic Alzheimer’s disease. Capitalizing on a large normative cohort and optimized imaging acquisition protocols, we highlight a biomarker of default mode network failure reflecting shared system-level pathophysiological mechanisms across aging and dysexecutive and amnestic Alzheimer’s disease and biomarkers of focal neurodegeneration reflecting distinct pathognomonic processes across the amnestic and dysexecutive Alzheimer’s disease phenotypes. These findings provide evidence that variability in inter-individual cognitive impairment in Alzheimer’s disease may relate to both modular network degeneration and default mode network disruption. These results provide important information to advance complex systems approaches to cognitive aging and degeneration, expand the armamentarium of biomarkers available to aid diagnosis, monitor progression and inform clinical trials.

## Graphical Abstract

**Graphical abstract:**
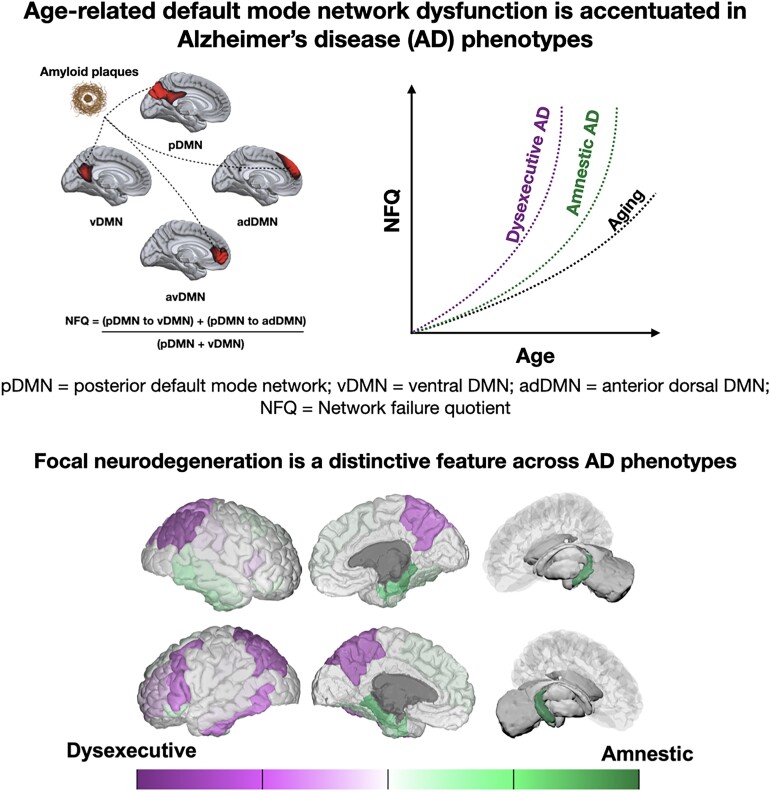

## Teaser

Corriveau-Lecavalier *et al*. show that age-related default mode network dysfunction is accrued in dysexecutive and amnestic Alzheimer’s disease. However, focal neurodegeneration is a distinctive feature across these phenotypes. This is relevant to advance complex systems approaches to cognitive aging and degeneration and therapeutic approaches targeting network pathophysiology.

## Introduction

Clinical syndromes arising from neurodegenerative diseases are thought to be caused by abnormal aggregates of proteins disrupting and damaging large-scale networks supporting specific cognitive abilities before expanding to the rest of the brain.^1-3^ In Alzheimer’s disease, the cascading network failure model^4,5^ proposes that the accumulation of beta amyloid plaques would accelerate the functional degradation of the default mode network (DMN) naturally occurring during the aging process.^6-10^ The disruption of this highly regulated homeostatic cycle would allow tau to expand within and between modular cognitive networks in an activity-dependent manner.^8,11-15^ Given that the spatial pattern of amyloid deposition is similar across Alzheimer’s disease phenotypes while patterns of neurodegeneration are phenotype-specific,^5,16-19^ the cascading failure network model hypothesizes that age- and disease-related functional disruption of the DMN would be common across Alzheimer’s disease phenotypes. In contrast, patterns of tau-driven neurodegeneration observed in modular networks would be phenotype-specific. This latter point is supported by the fact that the parieto-frontal network is selectively degenerated in the dysexecutive variant of Alzheimer’s disease,^18,20,21^ which is an atypical and relatively early-onset phenotype that initially and predominantly impairs core executive functions.^22^ This differs from the amnestic late-onset variant of Alzheimer’s disease, which is characterized by early neuronal loss in temporal areas underlying episodic memory.^23-26^

**Figure 1:**
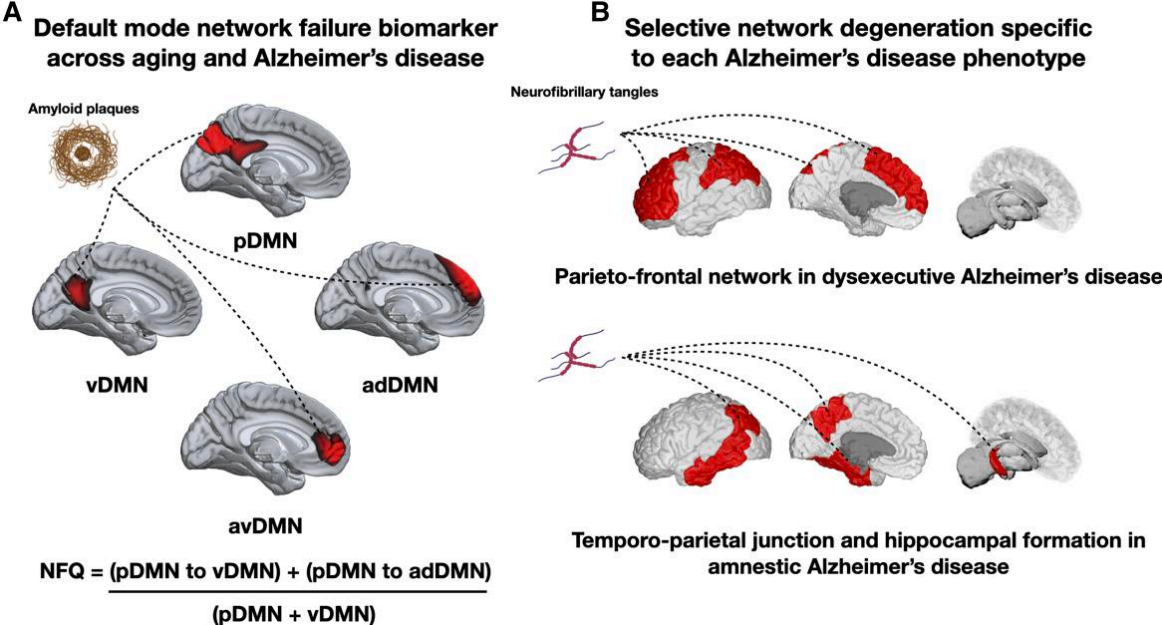
**Network biomarkers of amnestic and dysexecutive Alzheimer’s disease.** (**A**) Functional independent component analysis maps of subsystems forming the DMN. The NFQ is a single metric reflecting non-linear changes of the DMN in aging and Alzheimer’s disease. (**B**) Hypothesized areas of tau-driven neurodegeneration that are specific to the dysexecutive and amnestic Alzheimer’s disease. The parieto-frontal network, which is tightly linked to executive functioning, is selectively degraded in dysexecutive Alzheimer’s disease. Temporo-parietal areas, which to support episodic memory, are particularly vulnerable to neurodegeneration in amnestic Alzheimer’s disease. Brain rendering visualization was performed with MRIcroGL for the NFQ and BrainPainter^30^ for neurodegeneration markers. Amyloid plaques and tau tangles images were generated using BioRender (https://biorender.com/). NFQ = network failure quotient; DMN = default mode network; pDMN = posterior default mode network; vDMN = ventral DMN; adDMN = anterior dorsal DMN; avDMN = anterior ventral DMN.

If Alzheimer’s disease pathophysiology operates at the system level, macro-scale network biomarkers of shared and distinct pathophysiology across its clinical phenotypes are paramount to shed light on biological mechanisms underlying clinical heterogeneity, aid diagnosis and optimize clinical trials enrolment and design.^19^ Indeed, previous and current therapeutic interventions have almost exclusively focused on molecular and cellular physiology and these reductionist approaches have thus far failed to result in a significant clinical impact.^27^ This hints to the necessity to integrate multiple scales of brain organization to expand our understanding of Alzheimer’s disease pathophysiology from a complex system perspective. A disease model incorporating network physiology would allow to advance biomarker programmes aimed at the network-level physiology relevant to such a model and consequently targeting and monitoring the effects of network-level therapeutic strategies. We previously developed and validated a robust biomarker of DMN failure in Alzheimer’s disease, the network failure quotient (NFQ).^5,10^ The NFQ is thought to represent dynamic connectivity changes within and between DMN subnetworks (posterior DMN or pDMN; ventral DMN or vDMN; anterior dorsal DMN or adDMN) that occur in the aging process but that are accelerated in the context of Alzheimer’s disease.^9,10^ For instance, it has been previously demonstrated that pDMN connectivity decreases in aging and Alzheimer’s disease, as well as in *APOE4* carriers,^4,28^ leading to connectivity shifts from the pDMN to the vDMN and anterior dorsal adDMN.^4,5,9,29^ Accordingly, the NFQ is calculated by summing the median connectivity between the pDMN and ventral vDMN and between the pDMN and adDMN, which is then divided by the median connectivity between the pDMN and vDMN (see Fig. 1). Here, a higher NFQ value represents a greater functional connectivity disruption between the subcomponents of the DMN. The NFQ was found to linearly increase with rising levels of amyloidosis in the preclinical and clinical phases of Alzheimer’s disease at the group level,^5,10,31^ which may be due to the intimate relationship between amyloid precursor protein and synaptic activity,^32,33^ long-term potentiation and long-term depression in processing hubs of the brain forming the DMN.^34^ This biomarker is thus hypothesized to be a functional state marker of the DMN commonly observed across Alzheimer’s disease phenotypes,^5^ in the same way that amyloid-PET is.^5,19^ This is consistent with the known relationship between the DMN and amyloid deposition.^6,8^ Conversely, focal neurodegenerative markers are thought to be phenotype-specific and should therefore be of particular utility in understanding the macro-scale underpinnings underlying Alzheimer’s syndromic variability.^5,19,35^

This investigation stemmed from a collaborative effort between teams from Mayo Clinic and the Human Connectome Project-Aging (HCP-A). The overarching goal of this study is to investigate the shared and distinct macro-scale physiology across aging and amnestic and dysexecutive Alzheimer’s disease within the lens of the cascading network failure model and is divided into two main objectives. The first objective was to assess the robustness of the NFQ in a cohort covering a wide age spectrum and which was scanned using optimized functional MRI (fMRI) imaging protocols. To this end, we leveraged the breadth of the HCP-A dataset as a normative cohort of non-demented adults aged from 35 to over 100 years old to examine relationships between the NFQ and age, global and focal structural neurodegeneration markers, and cognition. The second objective was to assess the capacity of the NFQ and focal neurodegeneration markers to distinguish patients diagnosed with either the dysexecutive or amnestic variants of Alzheimer’s disease patients from the HCP-A cohort, as well as between Alzheimer’s disease phenotypes. Importantly, these comparisons were assessed at the patient level, which is an advancement compared with previous studies which solely relied on group-wise comparisons.^5,10^ Another essential facet of this study is that Alzheimer’s disease patients, just like HCP-A participants, were scanned using sequences used by the HCP-A, featuring high-resolution multi-echo structural images and longer resting-state connectivity acquisition time than to traditional protocols.

## Material and methods

### Participants

#### HCP-A normative cohort

**Figure 2:**
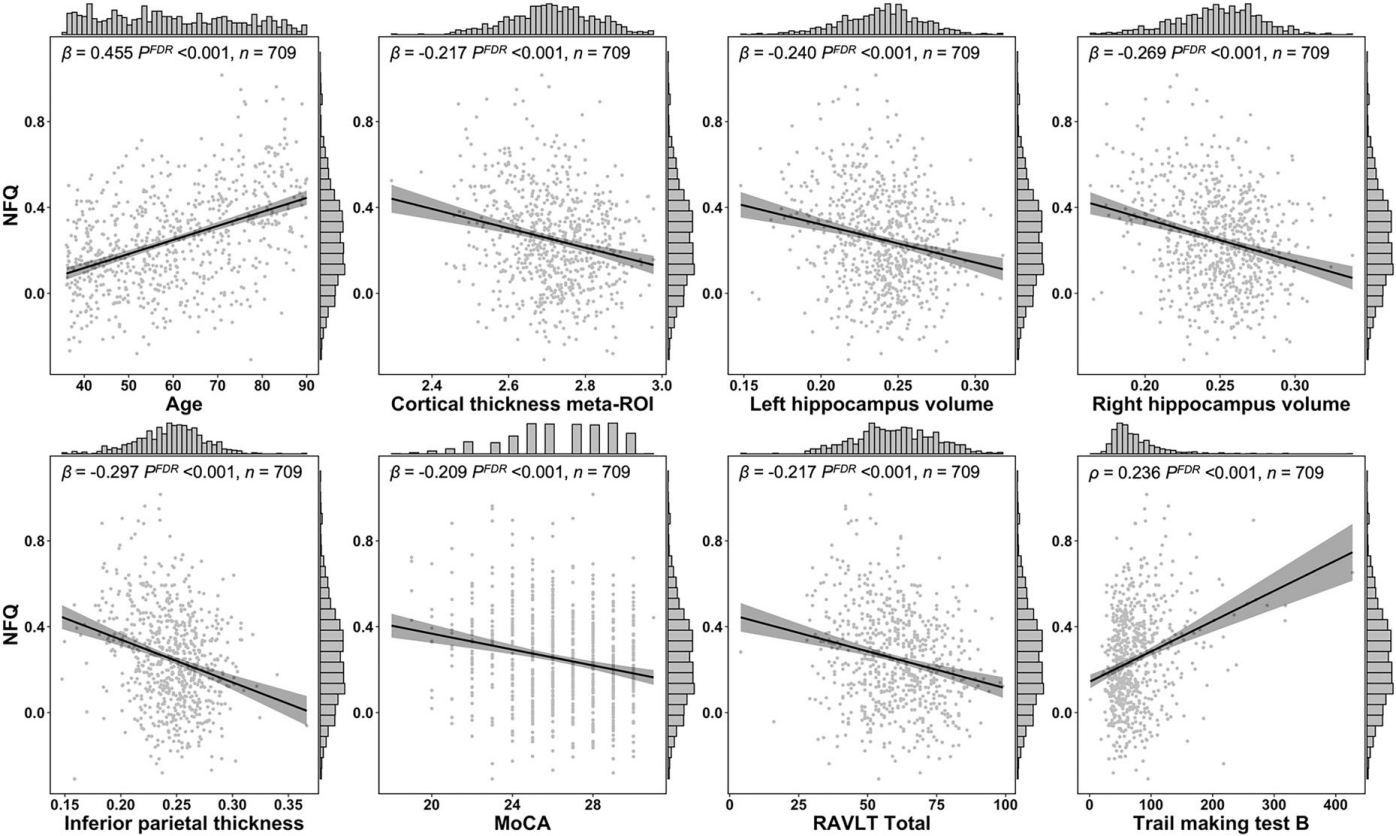
**Relationships between the NFQ, age, neurodegeneration markers and cognition in the HCP-A cohort.** Relationships were highly significant between the NFQ and age, cortical thickness as measured with a meta-ROI, hippocampi volume, inferior parietal thickness (summed across hemispheres) and cognitive performance (MoCA, RAVLT, Trail Making Test-B). NFQ = network failure quotient; ROI = region of interest; MoCA = Montreal Cognitive Assessment; RAVLT = Rey Auditory Verbal Learning Test; HCP-A = Human Connectome Project-Aging.

**Figure 3:**
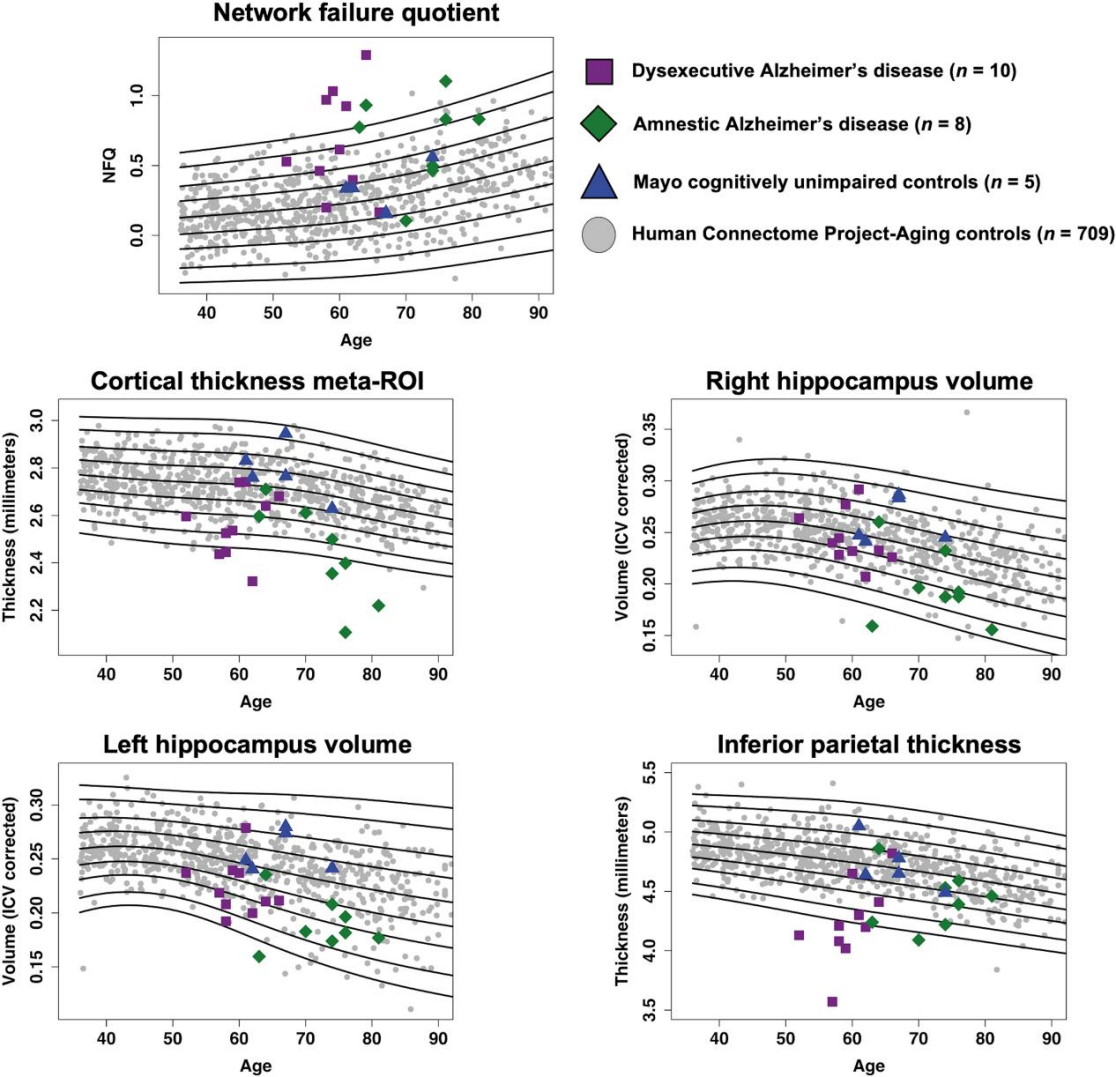
**Quantile curves on biomarkers of interest.** Age-adjusted curves are shown for centiles 0.4, 2, 10, 25, 50, 75, 90, 99, 99.6 for each biomarker (thick lines). dAD = dysexecutive Alzheimer’s disease; AD = Alzheimer’s disease; HCP-A = Human Connectome Project-Aging; ICV = intracranial volume; ROI = region of interest.

The HCP-A dataset (www.humanconnectomeproject.org) is thoroughly described in previous publications.^36,37^ Minimally processed structural MRI and resting-state fMRI images acquired at 3T were downloaded from the National Institute of Mental Health data archive (https://nda.nih.gov/) along with demographic and cognitive data (*N* = 724). Cognitive data included the Montreal Cognitive Assessment (MoCA),^38^ Trail Making Test-B (TMT-B)^39^ (time to completion) and the Rey Auditory Verbal Learning Test (RAVLT)^40^ (total score across five free recalls), which are tasks meant to assess global cognitive function, cognitive flexibility and episodic memory, respectively. Of note, Figs 2 and 3 only display participants up to age 90 in older to prevent individual identification of participants older than 90. Statistical analyses have however been computed with the actual age at visit.

#### Dysexecutive and amnestic Alzheimer’s disease cohorts

Patients with dysexecutive (*N* = 10) or amnestic (*N* = 8) Alzheimer’s disease were recruited at Mayo Clinic, Rochester, and enrolled in the present research project following their clinical visit in our tertiary behavioural neurology clinic. Dysexecutive patients met criteria for a progressive dysexecutive syndrome due to Alzheimer’s disease,^20^ meaning they presented with a predominant and progressive dysexecutive dementia syndrome for at least 6 months in the absence of predominant behavioural features (i.e. not meeting diagnostic criteria for the behavioural variant of frontotemporal dementia^41^), had positive biomarkers for Alzheimer’s disease pathology, and did not have a medical condition accounting for the clinical presentation. Amnestic patients met criteria for dementia due to Alzheimer’s disease^23^ in that they presented with a predominant and progressive amnestic dementia syndrome in the setting of positive biomarkers for Alzheimer’s disease pathology, and did not have an alternative clinical condition severe enough to account for the clinical presentation. Clinical diagnoses meeting inclusion criteria for either dysexecutive or amnestic predominant presentations were assigned prior to study enrolment by an experienced clinical behavioural neurologist after a clinical visit with the patient and an informant, a neurological examination and an extensive review of relevant clinical documentation. Alzheimer’s disease pathology was confirmed through PET imaging (see below).

#### Mayo Clinic cognitively unimpaired controls

We additionally recruited five cognitively unimpaired controls (referred to as ‘controls’) which were scanned at Mayo Clinic using the HCP-A protocol to assess potential systemic effects of site for the NFQ and structural MRI markers. Cognitive status was attributed prior to study enrolment with the same procedure described above. All controls were amyloid- and tau-PET negative (see below). This study was approved by the Mayo Clinic Institutional Review Board, and all participants or their designee provided written informed consent to participate in this study.

#### Image acquisition and processing

Structural and fMRI images for the HCP-A cohort and participants from Mayo Clinic (Alzheimer’s disease patients, controls) were collected with 3T Siemens Prisma scanners using identical acquisition sequences based on the HCP-A protocols, which are described in Harms *et al*.^37^ HCP-A participants were scanned across multiple sites (Massachusetts General Hospital or MGH, University of California, Los Angeles, or UCLA, University of Minnesota or UMinn, Washington University in St Louis or WashU), and Alzheimer’s disease patients and controls were scanned at Mayo Clinic, Rochester, MN.

#### Resting-state functional MRI

fMRI images were acquired in four different sessions each lasting 6min and 41 s, for a total of 488 volumes per session. Half of the sessions were acquired using an anterior-to-posterior sequence, while the other half was acquired using a posterior-to-anterior sequence, both in an interleaved fashion with a slice thickness of 2 mm. fMRI scans were acquired with a 2D multi-band (MB) gradient-recalled echo (GRE) echo-planar imaging (EPI) sequence, MB factor of 8 with a time of repetition (TR)/time of echo (TE) of 800/37 ms and a flip angle of 52°, and 2.9 mm isotropic voxels covering the whole brain (72 oblique axial slices).

Processing steps are thoroughly described in references.^4,10^ Briefly, they include removal of the first ten volumes, despiking using AFNI’s 3dDespike program (https://afni.nimh.nih.gov/), slice-timing correction followed by two pass realignment to the mean echo-planar image, co-registration to the structural image and normalization into the Mayo Clinic Adult Lifespan Template (https://www.nitrc.org/projects/mcalt/). High-dimensional independent components of interest from The Mayo Clinic Study of Aging connectivity Atlas^42^ (i.e. ventral, posterior, anterior ventral and anterior dorsal subparts of the DMN) (https://www.nitrc.org/frs/? group_id=1189) were transformed to individual space using the inverse warps created during segmentation and normalization. An anatomically based ‘noise region of interest (ROI)’ was derived for component-based correction by binarizing native-space cerebrospinal fluid and white matter segmentation at a 0.9 probability threshold and by eroding it by two voxels in each direction to avoid grey matter contamination. Voxel-wise time series were then extracted from this mask to be used in a principal component analysis. The first six principal components were combined with the six motion parameters and their first temporal derivatives to create a nuisance regression matrix for further processing. AFNI’s 3dBandpass program was used to detrend, simultaneously apply a band-pass filter set at 0.009–0.08 Hz and perform the nuisance regression using the nuisance regressor matrix. Simultaneous filtering and nuisance regression was done to avoid spectral misspecification of motion artefact further reducing the effect of motion confounds.^43^ The same program was used for time series variance normalization, masking and smoothing with a 8 mm at full-width half-maximum Gaussian kernel.

The NFQ was derived with a spatial–temporal regression within a multivariate framework incorporating all DMN subsystems of interest using the group independent component analysis (GICA) toolbox software package.^44^ A single summary of the NFQ was computed using a formula described in Wiepert *et al*.^10^ and shown in Fig. 1. This was done separately for all four resting-state scans for each participant, and metrics were then averaged within-participant across scanning sessions. We calculated the intra-class correlation coefficient for each DMN subsystem and the NFQ across scanning sessions to ensure signal consistency. This yielded excellent consistency for all measures of interest, with coefficients ranging from 0.84 to 0.86.^45^

Motion was accounted for at the scan level in the HCP-A cohort by only keeping scans with a frame-wise displacement lower than 0.25. This resulted in the removal of 56/2792 scans (15 participants). Of note, it was not possible to directly correct for motion in statistical analyses given that connectivity metrics were averaged across scanning sessions. This was rather accounted for in our processing pipeline, as noted above. Relationships between non-averaged NFQ metrics and motion (pitch, yaw, roll, translation, absolute movement, frame-wise movement) and physiologic (cardiac and respiratory pulse) parameters for the HCP-A cohort are nonetheless reported in Supplementary Table 1. In patients and Mayo controls, individual scans with clear excessive motions were discarded from analyses (>2 mm for XYZ translation, pitch, yaw, roll), resulting in the removal of 4/92 scans. All scans acquired at Mayo Clinic were also assessed using standardized quality control procedures by expert imaging analysts (M.K. and J.G.-R.) and were visually assessed by the first author (N.C.-L.) to ensure the absence of motion artefacts.

#### Structural MRI

Structural MRI images were acquired in a single session lasting 8min and 22 s using a multi-echo T1-weighted magnetization prepared rapid acquisition gradient echo sequence. Images were acquired using an interleaved anterior-to-posterior sequence with a slice thickness of 0.8 mm. The four echo times were 1.81, 3.6, 5.39 and 7.18 ms respectively, with a repetition time of 2500 ms, a flip angle of 8°, a field of view of 256×256 mm, and a voxel resolution of 0.8×0.8×0.8 mm.

Images were processed using standard FreeSurfer 7.1.1 pipeline.^46^ Volumetric and cortical thickness values were derived according to the Desikan-Killany atlas.^47^ A validated cortical thickness meta-ROI^48^ was calculated by averaging bilateral thickness estimates from the entorhinal cortex, inferior temporal lobe, medial temporal lobe, inferior parietal lobe, fusiform gyrus and precuneus. We additionally focused on two *a priori* brain regions, the hippocampus and the inferior parietal lobule. These regions were selected due to their selective degeneration in the dysexecutive^20,21,49^ and amnestic^50-52^ variants of Alzheimer’s disease as well as their involvement in episodic memory and executive functions, respectively.^53-57^ Hippocampi volumes were corrected for intracranial volume.

#### Amyloid and tau-PET imaging

PET images were acquired in Alzheimer’s disease patients only. Amyloid-PET and tau-PET were respectively acquired using Pittsburgh compound B (PiB) and ^18^Flortaucipir (AV1451) ligands produced using an on-site cyclotron. Processing steps for PET images are described in previous publications from our group.^58,59^ PET images were individually scaled to the cerebellar crus regions to yield region-wise standardized uptake value ratios (SUVRs). A global SUVR was derived from a validated meta-ROI for each patient. Thresholds for amyloid and tau positivity were set at >1.42 and >1.23, respectively.^59^

### Statistical analysis

Statistical analyses were performed using *R* (https://cran.r-project.org/) version 1.4. One-way ANOVAs with Tukey’s *post hoc* tests and *χ*^2^ analyses were conducted to assess between-group differences on continuous and categorical variables, respectively.

We first used a linear regression framework to assess relationships between the NFQ and age, cortical thickness as measured with the meta-ROI, left and right hippocampi volume, inferior parietal thickness (summed across hemispheres) and cognitive measures (MoCA, TMT-B, RAVLT). Variables of interest were normally distributed as per kurtosis and skewness measures (<1 in all cases), except for the TMT-B (skewness of 2.62 and kurtosis of 11.27). Thus, the Spearman method was chosen over Pearson for this score. False discovery rate correction was applied to *P*-values to account for multiple comparisons. We additionally computed mediation models using the mediation *R* package^60^ to assess whether the NFQ mediated the relationship between cognitive performance (episodic memory, cognitive flexibility) and volume/thickness of *a priori* selected regions putatively supporting these cognitive processes. A first model included the RAVLT total score as the dependent variable, hippocampal volume (summed across hemispheres) as the independent variable and the NFQ as the mediator. A second model included the TMT-B score as the dependent variable, inferior parietal thickness (summed across hemispheres) as the independent variable and the NFQ as the mediator. We assigned the NFQ as mediator based on the predictions from the cascading network failure model, which suggests that the NFQ reflects a global compensatory response to may attenuate detrimental effects of focal neurodegeneration on cognition. Effects were estimated using bootstrapping (10 000 simulations) and are expressed as quasi-Bayesian estimates with confidence intervals (CIs) set at 95%.

We next produced age-adjusted quantile curves for the NFQ, cortical thickness meta-ROI, left and right hippocampal volume and inferior parietal cortical thickness in the normative HCP-A cohort using the Generalized Additive Model for Location, Shape and Scale method (i.e. ‘gamlss’ *R* package).^61^ This method assesses various smoothing and distribution solutions and selects the best fit model based on the Global Deviance, Akaike Information Criterion and Schwartz Bayesian criterion. Model diagnostics were assessed with worm, Q–Q plots, and detrended transformed Owen’s plots. The final models were chosen based on these indices along with results of the model, residual diagnostics and visualization of the quantile curves. In a second step, we calculated age-adjusted centile ranks and *Z*-scores for participants scanned at Mayo (dysexecutive and amnestic Alzheimer’s disease patients and controls) based on the fitted quantile curve models. We assessed group differences between dysexecutive patients, amnestic patients, controls, and the normative HCP-A cohort on biomarkers of interest using one-way ANOVAs with Tukey’s *post hoc* tests on the age-adjusted *Z*-scores. We additionally conducted receiver operating curve (ROC) analyses on the age-adjusted *Z*-scores to distinguish between amnestic and dysexecutive Alzheimer’s disease patients groups (separately and combined) and the HCP-A cohort. Mayo controls were not involved in this latter analysis.

We performed an exploratory analysis by conducting separate pair-wise independent *t*-tests between dysexecutive patients, amnestic patients and the normative HCP-A cohort for the NFQ and focal markers of neurodegeneration that are part of the parieto-frontal network, the temporo-parietal junction and hippocampi. This was mainly done to yield Cohen’s *D* effect sizes to estimate the extent to which these metrics were able to distinguish dysexecutive and amnestic Alzheimer’s disease patients’ groups from the normative HCP-A cohort as well as to identify focal neurodegeneration biomarkers specific to each Alzheimer’s disease phenotype.

We also performed several supplementary analyses. First, we performed simple regression models between age and DMN subnetworks (pDMN, adDMN, vDMN) connectivity separately in the HCP-A cohort to assess how they compare with the NFQ. These analyses can be found in Supplementary Table 2 and showed that the NFQ related better with age than DMN subnetworks considered alone. Second, we repeated the quantile curve and group-wise ANOVA analyses on age-adjusted *Z*-scores on DMN subnetworks connectivity to assess how these metrics compared with the NFQ in distinguishing Alzheimer’s disease patients from controls/the HCP-A cohort. These analyses can be found in Supplementary Fig. 1 and Supplementary Table 3 and showed that the NFQ outperformed these metrics. Third, we assessed potential side effects by comparing age-adjusted *Z*-scores yielded by the quantile curve models on all biomarkers of interest (NFQ, cortical thickness ROI, left and right hippocampus volume, inferior parietal thickness) across all scanning sites (controls from Mayo Clinic; MGH, UCLA, UMinn, WashU). These analyses can be found in Supplementary Table 4 and showed that Mayo controls did not differ from other scanning sites on any of the metrics of interest.Results

## Results

### Demographics and Alzheimer’s disease biomarkers

Demographic, clinical, biomarker and cognitive data are listed in Table 1. There was no group difference in age or sex distribution. The dysexecutive cases trended towards a younger age given the association with this phenotype and younger onset Alzheimer’s disease.^20^ All patients with dysexecutive Alzheimer’s disease and all but one patient with amnestic Alzheimer’s disease were classified as A+/T+ based on PET imaging, according to the biological definition of Alzheimer’s disease.^62^ The amnestic patient classified as A+/T− based on PET imaging however had a tau-PET SUVR at <0.1 below the positivity cut-point, and cerebrospinal fluid (CSF) biomarkers obtained over the course of clinical care were consistent with Alzheimer’s disease pathology (Aβ_42_ < 487 pg/ml and *P*-tau > 58 pg/ml^63,64^). This patient was thus considered to be A+/T+ based on CSF biomarkers. Mayo Controls were all A−/T−.

**Table 1:** Demographic, clinical, cognitive and biomarker data

	HCP-A	dAD	AD	Mayo controls	*P*
Sample size	724	10	8	5	-
Age (median, Q1–Q3)	58.4 (47.2–72.4)	59.5 (58–61.8)	74 (68.5–76)	67 (62–67)	0.12
Sex (Male, Female)	319, 405	3, 7	6, 2	4, 1	0.09
MoCA (median, Q1–Q3)	26.5 (25–28)	9 (6.5–13)	11 (9–12)		<0.001^a^
Trail Making Test-A (median, Q1–Q3)	27.49 (21.22–36.19)				
Trail Making Test-B (median, Q1–Q3)	63.28 (48.07–89.07)				
RAVLT first trial (median, Q1–Q3)	5 (4–6)				
RAVLT total score (median, Q1–Q3)	60 (50.25–69.75)				
RAVLT list B (median, Q1–Q3)	5 (4–6)				
RAVLT short delay (median, Q1–Q3)	10 (7–12)				
Amyloid-PET SUVR (median, Q1–-Q3)		2.48 (2.07–2.61)	2.62 (2.41–2.81)	1.30 (1.30–1.31)	
Tau-PET SUVR (median, Q1–Q3)		2.14 (1.93–2.54)	1.89 (1.58–2.32)	1.21 (1.21–1.22)	

HCP-A = Human Connectome Project-Aging; dAD = dysexecutive Alzheimer’s disease; AD = amnestic Alzheimer’s disease; Q = quartile; MoCA = Montreal Cognitive Assessment; RAVLT = Rey Auditory Verbal Learning Test; SUVR = standard uptake value ratio.

a HCP-A & Controls > dAD & AD (both < 0.001).

### The network failure quotient relates with age and cognition in a large normative cohort of non-demented adults

We used a regression framework to assess relationships between the NFQ and age, cortical thickness, hippocampal volume and cognition in the HCP-A cohort. Summary results for regression analyses are listed in Table 2 and displayed in Fig. 2. The NFQ significantly correlated with age, cortical thickness meta-ROI, left and right hippocampus volume, inferior parietal thickness, and all measures of cognition (MoCA, RAVLT, TMT-B), with the strongest correlation being for age. All these models remained highly significant after correcting for multiple comparisons. A multivariate regression model with all variables of interest entered as predictors of the NFQ was highly significant [*F*(8, 695) = 27.3, *P* < 0.001, *R*^2^ = 0.243, $R_{\mathrm{adj}}^{2}$ = 0.234], with age, left hippocampus volume, right hippocampus volume and TMT-B remaining significant and independent predictors. Of note, a higher score on the TMT-B reflects of a poor performance as it indicates a longer time to complete the task, whereas a higher score on the RAVLT reflects a better performance, i.e. a higher number of words recalled. An inverse directionality in the relationships for these measures was therefore expected.

**Table 2:** Relationships between the network quotient failure, global and focal neurodegeneration markers and cognitive in the Human Connectome Project-Aging cohort

Simple regression models	*t*	*β*/*ρ*	*P* FDR	Adjusted *R^2^*	Lower CI	Upper CI
Age	13.60	0.455	<0.001	0.206	0.39	0.52
Cortical thickness meta-ROI	5.92	−0.217	<0.001	0.046	−0.29	−0.15
Left hippocampal volume	6.59	−0.240	<0.001	0.057	−0.31	−0.17
Right hippocampal volume	7.41	−0.269	<0.001	0.071	−0.34	−0.20
Inferior parietal thickness	8.26	−0.297	<0.001	0.087	−0.37	−0.23
MoCA	5.67	−0.209	<0.001	0.042	−0.28	−0.14
RAVLT total	5.84	−0.217	<0.001	0.046	−0.29	−0.14
TMT-B		0.326	<0.001		0.163	0.304

Multivariable regression framework	*t*	*β*	*P*	*R^2^*	Lower CI	Upper CI
Age	8.32	0.395	<0.001	0.16	0.30	0.49
Cortical thickness meta-ROI	0.16	0.007	0.871	0.000	−0.08	0.09
Left hippocampal volume	2.88	0.273	0.004	0.070	0.09	0.46
Right hippocampal volume	2.71	0.255	0.007	0.070	0.07	0.44
Inferior parietal thickness	1.36	−0.063	0.175	0.003	−0.15	0.03
MoCA	1.53	−0.063	0.127	0.003	−0.14	0.02
RAVLT total	0.90	0.038	0.368	0.001	−0.04	0.12
TMT-B	2.88	0.114	0.004	0.013	0.04	0.19

*β*/*ρ* and confidence intervals are standardised. FDR = false discovery rate; CI = confidence interval; ROI = region of interest; MoCA = Montreal Cognitive Assessment; RAVLT = Rey Auditory Verbal Learning Test; TMT-B = Trail Making Test-B.

The first model mediation model showed that the NFQ was a significant partial mediator in the relationship between hippocampal volume and scores on the RAVLT [indirect effect estimate of 9.52, CI (4.98–14.9), *P* < 0.001; direct effect: 66.96, CI (48.43–85.7), *P* < 0.001; proportion mediated: 12.29%, *P* < 0.001]. The second model demonstrated that the NFQ was a significant partial mediator in the relationship between inferior parietal thickness and scores on the TMT-B [indirect effect estimate of −14.78, CI (−21.81–−8.62), *P* < 0.001; direct effect: −33.00, CI (−50.09–−15.65), *P* < 0.001; proportion mediated: 30.8%, *P* < 0.001].

### The network failure quotient is a common biomarker across Alzheimer’s disease phenotypes

Quantile curves and group-wise comparisons results for the NFQ and neurodegeneration biomarkers of interest are displayed in Figs 3 and 4 respectively, and individual centile ranks and *Z*-scores for dysexecutive and amnestic Alzheimer’s disease patients and Mayo controls are listed in Table 3. All diagnostic plots can be found in Supplementary Figs 2–16.

**Figure 4:**
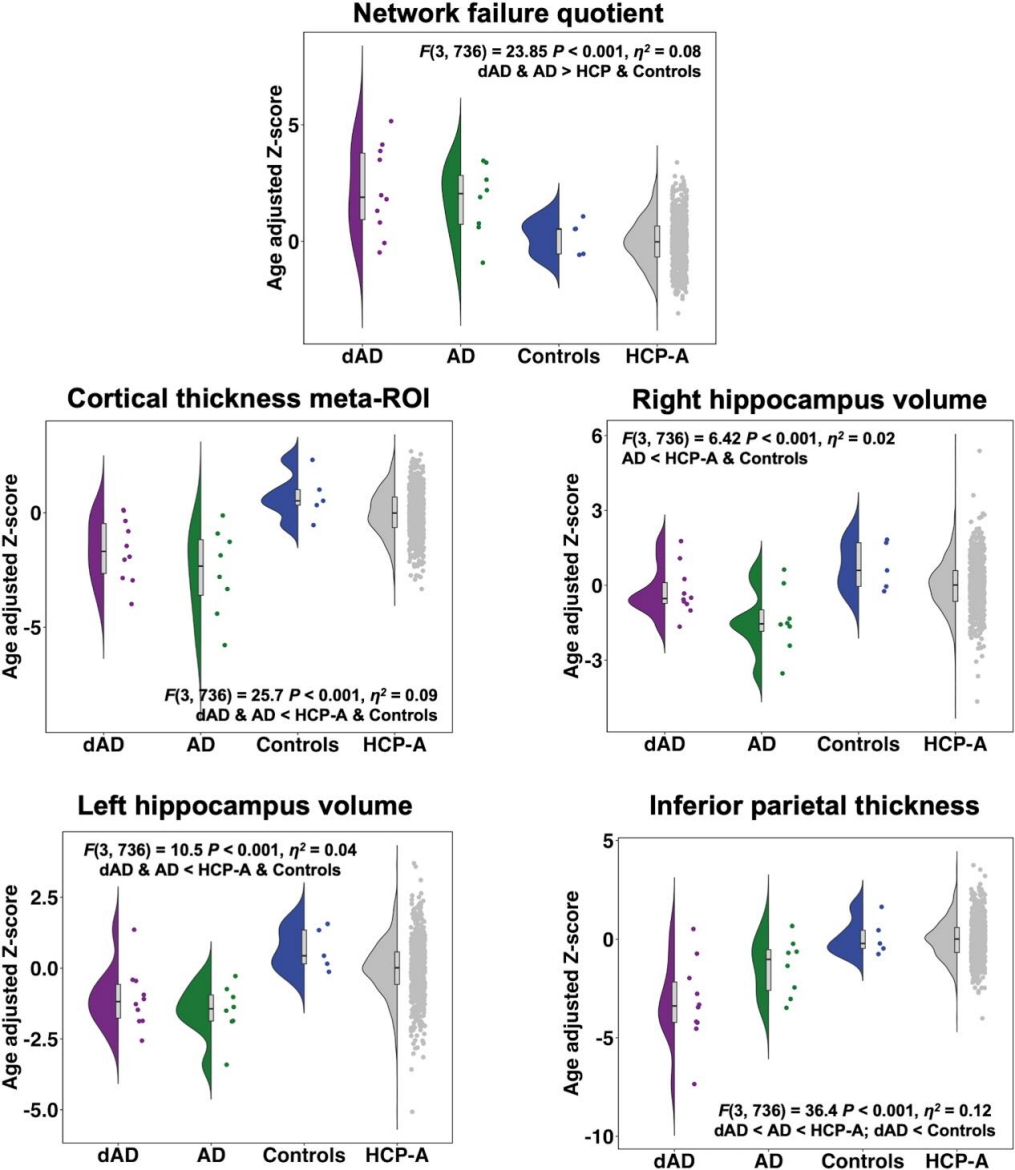
**Group-wise comparisons on age adjusted *Z*-scores.** *Z*-scores were derived from centile curve models for each biomarker of interest. NFQ = network failure quotient; ICV = intracranial volume; ROI = region of interest.

**Table 3:** Centile ranks and *Z*-scores for biomarkers in amnestic and dysexecutive Alzheimer’s disease patients and Mayo clinic cognitively unimpaired controls

	Network failure quotient		Cortical thickness meta-ROI		Left hippocampus volume		Right hippocampus volume		Inferior parietal thickness	
Patient	*Z*-score	Rank	*Z-*score	Rank	*Z*-score	Rank	*Z*-score	Rank	*Z*-score	Rank
dAD1	4.15	1.00	−1.93	0.03	−0.41	0.34	1.08	0.86	−4.54	0.00
dAD2	−0.47	0.32	−0.36	0.36	−1.10	0.14	−0.64	0.26	0.52	0.70
dAD3	3.88	1.00	−2.86	0.00	−2.55	0.01	−1.01	0.16	−4.23	0.00
dAD4	0.81	0.79	−4.01	0.00	−1.87	0.03	−1.65	0.05	−3.31	0.00
dAD5	5.16	1.00	−0.81	0.21	−1.27	0.10	−0.50	0.31	−1.97	0.02
dAD6	3.50	1.00	0.12	0.55	1.35	0.91	1.77	0.96	−2.77	0.00
dAD7	1.98	0.98	0.09	0.54	−0.46	0.32	−0.75	0.23	−0.73	0.23
dAD8	1.81	0.97	−1.46	0.07	−0.94	0.17	0.25	0.60	−4.19	0.00
dAD9	1.31	0.90	−2.96	0.00	−1.47	0.07	−0.57	0.29	−7.36	0.00
dAD10	−0.07	0.47	−2.06	0.02	−1.86	0.03	−0.33	0.37	−3.45	0.00
AD1	2.20	0.99	−2.80	0.00	−1.04	0.15	−1.51	0.07	−0.23	0.41
AD2	3.46	1.00	−5.77	0.00	−1.54	0.06	−1.34	0.09	−1.35	0.09
AD3	0.61	0.73	−1.87	0.03	−0.75	0.23	0.08	0.53	−2.45	0.01
AD4	0.77	0.78	−3.34	0.00	−1.93	0.03	−1.65	0.05	−0.69	0.24
AD5	2.65	1.00	−1.27	0.10	−3.42	0.00	−3.53	0.00	−3.03	0.00
AD6	3.38	1.00	−0.12	0.45	−0.28	0.39	0.63	0.73	0.67	0.75
AD7	1.90	0.97	−4.39	0.00	−1.43	0.08	−2.41	0.01	−0.63	0.27
AD8	−0.91	0.18	−0.92	0.18	−1.89	0.03	−1.56	0.06	−3.47	0.00
CU1	1.07	0.86	−0.54	0.29	0.41	0.66	0.58	0.72	−0.92	0.18
CU2	0.53	0.70	0.33	0.63	−0.19	0.43	−0.26	0.40	−0.71	0.24
CU3	0.54	0.70	1.01	0.84	0.11	0.54	−0.06	0.47	1.69	0.95
CU4	−0.53	0.30	0.52	0.70	1.58	0.94	1.71	0.96	0.34	0.63
CU5	−0.57	0.29	2.31	0.99	1.35	0.91	1.84	0.97	−0.42	0.34

dAD = dysexecutive Alzheimer’s disease; AD = amnestic Alzheimer’s disease; CU = cognitively unimpaired (Mayo controls). ROI = region of interest.

After assessing of the smoothing and transformation parameters for the NFQ, the best fit model used a penalized spline smoothing method and a normal distribution for the calculation of the centiles. Model diagnostics showed a normal distribution of the residuals around zero, as well as the density and Q−Q plots. Quantile residuals plot showed a mean close to zero, a coefficient of skewness of <1 and a coefficient or kurtosis of 2.94. Visualization of the worm and detrended transformed Owen’s plots showed appropriate fitting of variance and a normal distribution of residuals.

Centile ranks for the NFQ show that both dysexecutive and amnestic patients differed highly from the HCP-A normative cohort, with 7 out of 10 dysexecutive (70%) and 5 out of 8 amnestic (64%) patients being ranked above the 90th centile. Of the remaining patients, one dysexecutive (79th) and two amnestic (73th and 79th centile) were above the median, while two dysexecutive (32nd and 47th centile) and one amnestic (18th centile) fell below the median. Group-wise comparisons on age-adjusted *Z*-scores showed that both the dysexecutive and amnestic patient groups differed from the HCP-A normative cohort and Mayo controls but could not be distinguished from each other. The area under the curve (AUC) was 84.2% for dysexecutive Alzheimer’s disease (CI 68.7–99.6%), 83% (CI 63.7–100%) for amnestic Alzheimer’s disease when compared with the HCP-A cohort separately, and 83.7% (CI 72–95.4%) when combined.

Pair-wise comparisons on raw NFQ values revealed large effect sizes for the dysexecutive and amnestic Alzheimer’s disease groups when separately compared with the HCP-A normative cohort, and significance of these comparisons survived correction for multiple comparisons (see Fig. 4). However, the NFQ did not discriminate between phenotypes: the group comparison was not statistically significant (*P* = 0.76).

### Focal degeneration markers are specific to each Alzheimer’s disease phenotype

For all neurodegeneration markers, models retained used a penalized spline smoothing method and a normal distribution for the calculation of the centiles. Model diagnostics show the plots of residuals are normally distributed around zero, as well as the density and Q−Q plots. Quantile residuals showed means close to zero and skewness coefficients <1 in all cases, and kurtosis coefficients varying between 2.9 and 4.8. Visualization of the worm and detrended transformed Owen’s plots showed appropriate fitting of variance and a normal distribution of residuals for all variables.

Centile ranks for the inferior parietal thickness revealed that eight out of ten dysexecutive patients were ranked in the second centile or lower, whereas only three out of eight amnestic patients were in that range. The AUC was 90.1% (CI 75.7–100%) for dysexecutive Alzheimer’s disease and 77.8% (CI 59.3–96.3%) for amnestic Alzheimer’s disease when compared with the HCP-A cohort separately, and 84.7% (CI 73.1–96.2%) when combined. In contrast, six amnestic patients had right hippocampus volume in the 10th centile or lower, whereas it was only the case for one dysexecutive patient. The AUC was 59.6% (CI 40.3–78.8%) for dysexecutive Alzheimer’s disease, 80.7% (CI 60.6–100%) for amnestic Alzheimer’s disease and 68.9% (CI 54.4–83.3%) when patient groups were combined. Results for the left hippocampus volume showed less discrepancy between patient groups, where five dysexecutive and five amnestic patients were in the 10th centile or lower. The AUC was 79.5% (CI 62.5–96.5%) for dysexecutive Alzheimer’s disease, 88.8% (CI 80.9–96.6%) for amnestic Alzheimer’s disease and 83.6% (CI 73.5–93.7%) when patient groups were combined. Cortical thickness as assessed with the meta-ROI showed similar results, with six dysexecutive and six amnestic patients in the 10th centile or lower. The AUC was 82.3% (CI 68.5–96.1%) for dysexecutive Alzheimer’s disease, 90.2% (CI 79.1–100%) for amnestic Alzheimer’s disease and 85.8% (CI 76.7–94.9%) when patient groups were combined.

Consistent with the analyses described above, group comparisons on age-adjusted *Z*-scores showed that inferior parietal thickness significantly differed across groups, where dysexecutive patients had lower thickness compared with amnestic patients, controls and the HCP-A normative cohort, and amnestic patients had lower thickness compared with the HCP-A normative cohort but not controls. Right hippocampus volume was smaller in amnestic patients compared with both the HCP-A normative cohort and controls, while dysexecutive patients did not differ from other groups. Both dysexecutive and amnestic patients’ groups had lower cortical thickness as measured with the meta-ROI and lower left hippocampus volume compared with the HCP-A normative cohort and controls but did not differ from each other.

**Figure 5:**
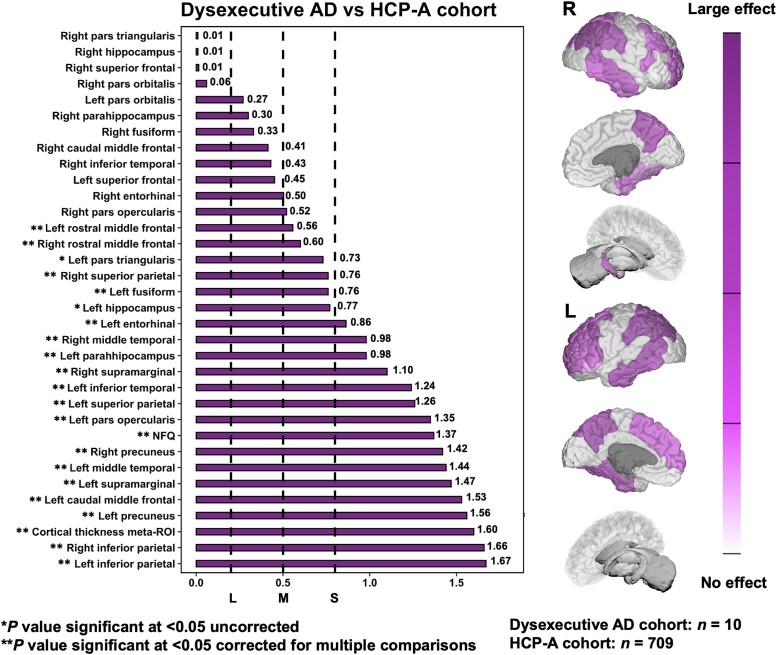
**Cohen’s *D* effect sizes yielded by pair-wise comparisons for focal neurodegeneration biomarkers and the NFQ in dysexecutive Alzheimer’s disease versus HCP-A.** Brain renderings depict a gradation of effect sizes for focal neurodegeneration in dysexecutive Alzheimer’s disease ranging from large to small/no effect. Cohen’s D effect sizes were considered small, medium and large at 0.3, 0.5 and 0.8, respectively (dashed lines). AD = Alzheimer’s disease; HCP-A = Human Connectome Project-Aging; ROI = region of interest; NFQ = network failure quotient.

**Figure 6:**
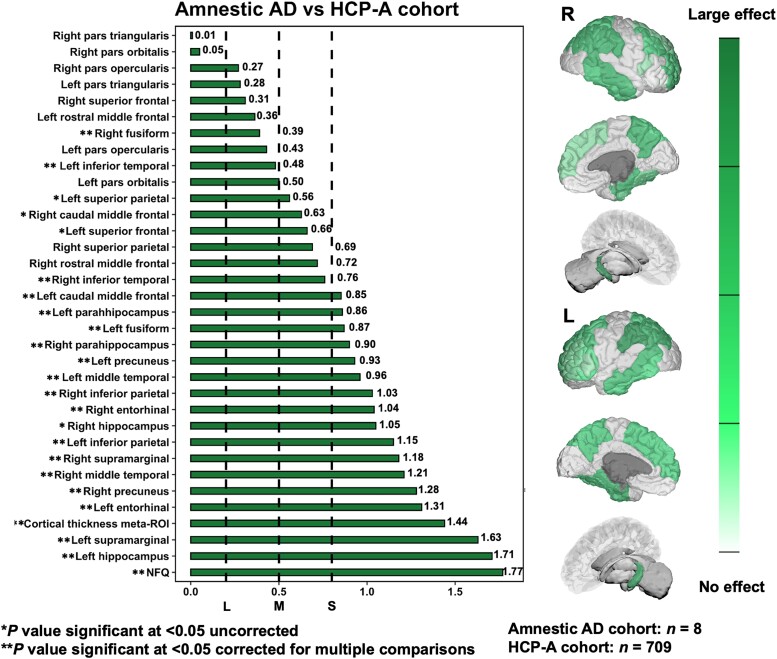
**Cohen’s *D* effect sizes yielded by pair-wise comparisons for focal neurodegeneration biomarkers and the NFQ in amnestic Alzheimer’s disease versus HCP-A.** Brain renderings depict a gradation of effect sizes for focal neurodegeneration in amnestic Alzheimer’s disease ranging from large to small/no effect. Cohen’s D effect sizes were considered small, medium and large at 0.3, 0.5 and 0.8, respectively (dashed lines). AD = Alzheimer’s disease; HCP-A = Human Connectome Project-Aging; ROI = region of interest; NFQ = network failure quotient.

**Figure 7:**
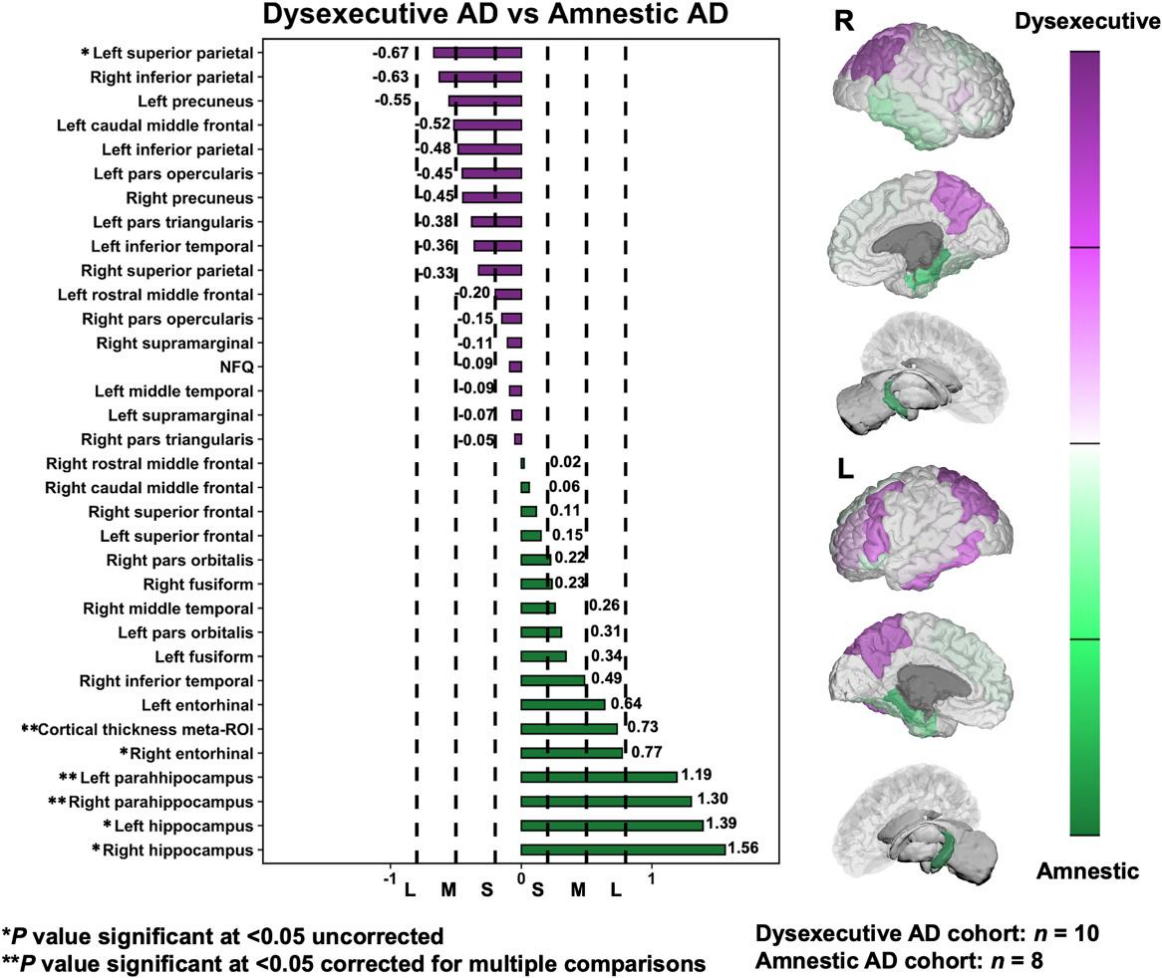
**Cohen’s D effect sizes yielded by pair-wise comparisons for focal neurodegeneration biomarkers and the NFQ in dysexecutive versus amnestic Alzheimer’s disease.** Brain renderings depict a gradation of effect sizes for focal neurodegeneration specific to dysexecutive or amnestic Alzheimer’s disease. Cohen’s *D* effect sizes were considered small, medium and large at 0.3, 0.5 and 0.8, respectively (dashed lines). AD = Alzheimer’s disease; ROI = region of interest; NFQ = network failure quotient; S = small; M = medium; L = large.

We then performed pair-wise comparisons between dysexecutive and amnestic Alzheimer’s disease phenotypes and the HCP-A normative cohort to yield Cohen’s *D* for focal neurodegeneration markers. When comparing dysexecutive patients to the HCP-A normative cohort, significant and largest effects sizes were found for inferior parietal areas, followed by parieto-frontal and temporal areas with a predominance towards the left hemisphere (see Fig. 5). When comparing amnestic patients to the HCP-A normative cohort, significant and largest effect sizes mostly involved temporo-parietal areas bilaterally, including the hippocampi and entorhinal cortices (see Fig. 6). Direct comparisons between Alzheimer’s disease phenotypes revealed a significant effect for the left superior parietal lobule being preferentially degenerated in dysexecutive patients, which was characterized by a medium effect size (see Fig. 7). It is interesting to note that other regions with the highest effect sizes for this group exclusively involved parieto-frontal areas, although they did not reach statistical significance. Conversely, regions showing significant effects for amnestic patients involved hippocampal and parahippocampal areas bilaterally with large effect sizes (ranging from 1.19 to 1.56), followed by the right entorhinal and the cortical thickness meta-ROI with relatively smaller effect sizes.

## Discussion

We first demonstrated that the NFQ related with age, structural markers of neurodegeneration and cognition in a large normative cohort of non-demented adults, thus showing that this biomarker of DMN dysfunction captures age-related changes in the structure of the brain and cognition. We then delineated biomarkers of DMN dysfunction and focal neurodegeneration reflecting shared and distinct system-level pathophysiology of dysexecutive and amnestic Alzheimer’s disease. High NFQ values were common across both the dysexecutive and amnestic manifestations of the disease. In contrast, focal markers of neurodegeneration were phenotype-specific, where areas from the parieto-frontal network were selectively degraded in dysexecutive patients and temporal areas were specifically degenerated in amnestic patients. These findings are in large agreement with the cascading network failure model, are important for unravelling the biological mechanisms underlying the syndromic variability of Alzheimer’s disease, prompt for studies in larger patient cohorts, and have potentially meaningful clinical and therapeutic implications.

### Network failure quotient across the age spectrum

We first assessed NFQ’s robustness as a biomarker of DMN failure in aging by assessing its relationships with age, structural markers of degeneration (hippocampi volume, cortical thickness meta-ROI, inferior parietal thickness) and cognition (MoCA, RAVLT, TMT-B) in a large normative cohort of non-demented adults. We found significant relationships between the NFQ and all aforementioned variables. The correlation between NFQ and age likely reflects gradual and progressive non-linear connectivity changes known to occur within and between DMN subsystems occurring in aging as previously documented,^9,65^ although to a lesser extent than what is observed in Alzheimer’s disease.^9,65,66^ The present study is however novel in that we used a normative cohort covering a wider age range and with an optimized resting-state fMRI imaging protocols compared with previous studies from our group conducted in the Mayo Clinic Study of Aging and Alzheimer’s Disease Neuroimaging Initiative cohorts.^10,67^ This provides additional construct validity to the concept of the NFQ as a system-level biomarker of DMN failure relating to neurodegeneration and cognition markers across the aging spectrum. Another new and interesting finding is the significant relationship between the NFQ and inferior parietal thickness, i.e. a brain region linked to executive functioning^53^ that is significantly degraded in dysexecutive Alzheimer’s disease. This is consistent with the finding of a significant relationship between the NFQ and performance on the TMT-B, which assesses cognitive flexibility and is particularly impaired in this syndrome.^20^ Moreover, we found that the NFQ was a significant partial mediator of the relationship between episodic memory and cognitive flexibility performance and volume/thickness of regions putatively supporting these cognitive processes. It is thus possible that the NFQ represents a marker of global efficiency, as suggested by previous work from our group conducted in the MCSA cohort.^10^ This is consistent with the current observation that the NFQ tracks with neurodegeneration and cognitive impairment not only in the amnestic variant of the disease but also in its dysexecutive presentation. It is important to keep in mind that although the NFQ significantly related with age, structural markers of the brain and cognition, the error margins around the fit were quite large and the effect sizes were in the small to moderate range (especially for structural markers and cognition), indicating substantial variability.

### Default mode network failure and focal neurodegeneration across Alzheimer’s disease phenotypes

When assessing the NFQ’s capacity to distinguish Alzheimer’s disease patients from the HCP-A normative cohort, we found that this biomarker was a highly distinctive and common feature of both Alzheimer’s disease phenotypes at the individual and group levels. This agrees with predictions from the cascading network failure model,^4,5^ which positions hubs forming the DMN as key modulators coordinating global functional compensatory responses associated with amyloid precursor protein processing involved with synaptic activity,^32,33^ long-term potentiation and long-term depression.^34^ This chronic hyperconnectivity occurring in areas known to accumulate amyloid^8,10,68^ results in higher NFQ values across Alzheimer’s disease clinical presentations.^49^ These findings provide evidence for the NFQ as a promising biomarker of the system-level pathophysiology of Alzheimer’s disease related to aberrant amyloid-processing within the DMN. These functional changes would likely occur prior to amyloid plaque formation as suggested by our group-level analyses in non-demented adults together with our prior work in individuals in the preclinical phase of the disease using lower quality fMRI sequences,^10^ and during the symptomatic phase after amyloid plaque deposition as supported by our findings at the individual level in Alzheimer’s disease patients. Of note, age-adjusted NFQ values were below the median of the HCP-A cohort for two dysexecutive and one amnestic patients. While the cause behind these low values is uncertain, this could be due to technical factors or non-degenerative biological factors (e.g. vascular or metabolic disease).

In contrast to the NFQ, focal markers of neurodegeneration distinguished between the dysexecutive and amnestic phenotypes of Alzheimer’s disease. Areas yielding the largest effect sizes in dysexecutive patients either compared with the HCP-A normative cohort or amnestic patients were mainly situated in the canonical parieto-frontal network, which is the macro-scale underpinning of executive functions.^53,54,69,70^ Areas typically associated with episodic memory capacities and known to be early sites of tau accumulation in amnestic Alzheimer’s disease, namely the hippocampus, parahippocampus and entorhinal cortex,^71-74^ showed selective neurodegeneration in amnestic patients. These results join extant literature suggesting that Alzheimer’s disease phenotypes are indiscernible at the micro-scale molecular level but can be distinguished based on patterns of macro-scale dysfunction, neurodegeneration and tau accumulation.^16,18,21,49,75-77^ This supports the idea of macro-scale network degeneration as an endophenotype between accumulation of pathological proteins aggregates, tau in this particular instance, and inter-individual variability in cognitive impairment in neurodegenerative disease.^2,19^ The reasons as to why certain macro-scale networks are selectively vulnerable to degeneration across Alzheimer’s disease phenotypes remain largely elusive. Studies have suggested shared genetic expression,^78,79^ cortical microstructure^80,81^ and molecular regional factors^82,83^ within and between canonical large-scale networks as potential explanations, but further research is still needed.

### Clinical and therapeutic implications

The development of system-level biomarkers of shared and distinct pathophysiology across Alzheimer’s disease phenotypes has diverse implications spanning mechanistic insights and clinical endeavours. Current Alzheimer’s disease biomarkers measured through blood plasma, CSF or PET imaging do not measure relevant network integrity and usually reach a plateau over the course of the disease. The NFQ is thought to rise in the preclinical phase of Alzheimer’s disease, prior to the onset of symptom and even before detectable molecular events assessed through PET-imaging.^4,10^ This suggests that the NFQ is a potential target for a diagnostic biomarker to detect Alzheimer’s disease in its earliest stages and as a potential herald of future cognitive decline in prospective studies. This is currently possible at the group level, and additional study is required to use large-scale network biomarkers at the single-subject level in the preclinical phase. This contrasts with the clinical phase in which individual subject biomarker performance is more robust. We however acknowledge that advanced protocols such as those used by the HCP-A pose feasibility challenges for implementation in clinical settings, at least for now. Regardless, our results highlight the potential of non-invasive functional biomarkers and encourage their adaptation to clinical settings. This could be done, for instance, by developing shortened but equally robust fMRI scanning protocols or assessing metrics aligned with the NFQ using electroencephalography. Network degeneration, although occurring later in disease course compared with functional abnormalities,^84^ has potential to aid in differential diagnostic and predict cognitive trajectories. This is particularly important in the context of the recent characterization of dysexecutive Alzheimer’s disease, which is often mistaken for the amnestic form of the disease or confused with other progressive dysexecutive syndromes.^85-87^ Network biomarkers are also paramount to guide the development of new therapeutic interventions. Such information could help enriching biological stratification prior to enrolment in clinical trials or develop treatments targeting large-scale brain systems in preclinical and clinical disease stages such as drug treatments reducing hyperactivity of disease-relevant networks as previously tested in amnestic mild cognitive impairment^88,89^ or transcranial magnetic stimulation as seen in multiple sclerosis (for a review see^90^). This latter point is far from trivial, as therapeutic interventions from the last decades, focusing solely on molecular and cellular physiology and generally conducted in patient with advanced clinical impairment, have failed to provide sorely needed treatment in humans.^27^ Knowledge about patterns of phenotype-specific network neurodegeneration may also inform design of clinical trials by adjusting desired biological and clinical endpoints according to phenotype-specific symptomatology, hereby facilitating the inclusion of patients of atypical forms of the disease.^19,91^

### Strengths and limitations

A strength of this study is the use of the HCP-A dataset as a normative cohort. The inclusion of a large normative cohort is of particular interest to assess the system-level pathophysiologic processes at the individual level. This allowed us to provide individualized age-adjusted metrics for the NFQ as well as focal neurodegeneration markers for dysexecutive and amnestic Alzheimer’s disease patients instead of solely relying on group-wise comparisons. It will be crucial to keep leveraging large normative cohorts with optimal acquisition protocols to assess disease pathophysiology at the individual level and meaningfully assess individual risk of developing Alzheimer’s disease and intervene accordingly. Another strength of this study is that our Alzheimer’s disease patients were deeply characterized at the clinical and imaging levels, and they were scanned with HCP-A protocols,^36,37^ making it a unique clinical dataset with optimized, state-of-the-art fMRI data. This is particularly important in the context of a study involving fMRI, where prolonged acquisition times may be important to detect signal in brain connectivity relevant to disease pathophysiology.^92^

Several limitations inherent in this study must be considered. Cohorts of dysexecutive and amnestic Alzheimer’s disease patients were considerably small compared with studies examining similar research questions. Studies in larger patient groups are necessary to support the claims made in this study about the clinical utility of measures of functional brain aging such as the NFQ and for the advancement of therapeutic programmes aimed at the macro-scale physiology of the brain. Although we used a large, deeply sampled normative cohort, it remains far too small for the use of quantile curve analyses aimed at establishing normative charts to be used in clinical practice. We rather used quantile curves analyses to yield NFQ values adjusted for age and to present our results in a clear, interpretable way. The cross-sectional nature of our design prevents assessing longitudinal trajectories, which would be useful to identify inflection points over the disease course when these biomarkers might be optimal to identify Alzheimer’s disease and distinguish between its clinical manifestations. All Alzheimer’s disease patients were recruited at the stage of dementia. A crucial next step would be to assess how and when functional and structural network biomarkers described in this study manifest in the preclinical phase of Alzheimer’s disease, prior to the emergence of significant cognitive symptomatology. Finally, the HCP-A cohort does not include PET imaging or fluid biomarkers, and henceforth we could not ascertain the presence or absence of underlying Alzheimer’s disease pathology in participants from that cohort.

## Concluding remarks

In summary, we highlighted biomarkers of global network failure and focal neurodegeneration reflecting shared and distinct system-level pathophysiology across aging and dysexecutive and amnestic Alzheimer’s disease phenotypes at the group and individual levels. Amyloid-related disruption of the DMN is a highly distinctive and common feature across Alzheimer’s disease phenotypical presentations, whereas syndromic variability is seemingly explained by neurodegeneration of vulnerable modular networks, in line with the cascading network failure model^4,5^ and a global information processing model of neurodegeneration.^93^ These findings have clear relevance to elucidate the heterogeneity of Alzheimer’s disease and have implications for its diagnosis and treatment, although pending replication in larger patient cohorts.

## Supplementary Material

fcad058_Supplementary_Data

## Data Availability

The HCP-A cohort is a publicly available dataset (https://www.humanconnectome.org/study/hcp-lifespan-aging). Mayo Clinic data can be shared upon reasonable request to the corresponding author and will be made available as part of the HCP-A dataset.

## Abbreviations

adDMN =: anterior dorsal DMN
AV1451 =: ^18^Flortaucipir
CI =: confidence interval
DMN =: default mode network
EPI =: echo-planar imaging
GICA =: group independent component analysis
GRE =: gradient-recalled echo
HCP-A =: Human Connectome Project-Aging
MB =: multi-band
MoCA =: Montreal Cognitive Assessment
MPRAGE =: magnetization prepared rapid acquisition gradient echo
NFQ =: Network failure quotient
pDMN =: posterior DMN
PiB =: Pittsburgh B Compound
RAVLT =: Rey Auditory Verbal Learning Test
ROC =: receiver operating curve
ROI =: region of interest
SUVR =: standardised uptake value ratio
TE =: time of echo
TR =: time of repetition
TMT-B =: Trail Making Test-B
UCLA =: University of California, Los Angeles
UMinn =: University of Minnesota
vDMN =: ventral DMN
WashU =: Washington University in St Louis.
