# Supplementary material for: Default mode network failure and neurodegeneration across aging and amnestic and dysexecutive Alzheimer’s disease
Source: Brain Commun. 2023 Mar 8;5(2):fcad058. doi: 10.1093/braincomms/fcad058 (PMC10066575; doi:10.1093/braincomms/fcad058)
Supplement: fcad058_Supplementary_Data [file fcad058_supplementary_data.pdf]

Supplementary Table 1 Variance shared between the network failure quotient and covariates of non-interest in the Human Connectome Project-Aging cohort

Motion parameters	Translation X	Translation Y	Translation Z	Pitch	Roll	Yaw	Absolute movement	Framewise movement
pDMN median	0.017	0.022	0.017	0.001	0.011	0.020	0.011	0.014
adDMN median	0.004	0.001	0.000	0.001	0.000	0.001	0.000	0.000
vDMN median	0.000	0.001	0.000	0.000	0.000	0.001	0.000	0.001
NFQ	0.058	0.053	0.051	0.039	0.045	0.059	0.049	0.061

Biological parameters	Cardiac pulse			Respiratory oximetry pulse		
	Median	SD	IQR	Median	SD	IQR
pDMN median	0.001	0.008	0.001	0.004	0.012	0.010
adDMN median	0.000	0.002	0.000	0.004	0.000	0.000
vDMN median	0.003	0.002	0.002	0.000	0.005	0.001
NFQ	0.000	0.027	0.006	0.004	0.022	0.019

Values are expressed in R^2 yielded by Pearson correlations. DMN = Default mode network pDMN = Posterior DMN; adDMN = Anterior dorsal DMN; avDMN = Anterior ventral DMN; vDMN = Ventral DMN; NFQ = Network failure quotient; SD = Standard deviation; IQR = Interquartile range.

Supplementary Table 2 Relationships between the network quotient failure and default mode subnetworks and age in the Human Connectome Project-Aging cohort

Variable	*t*	*β*	*P*	Adjusted R^2
NFQ	13.92	0.460 (0.40; 0.52)	< .001	0.210
pDMN	13.2	-0.441 (-051; -0.38)	< .001	0.193
adDMN	4.23	-0.155 (-0.23; -0.08)	< .001	0.023
vDMN	8.32	-0.296 (-0.37; -0.23)	< .001	0.086

NFQ = Network failure quotient; DMN = Default Mode Network; pDMN = Posterior DMN; adDMN = Anterior dorsal DMN; vDMN = Ventral DMN. Confidence intervals for beta values are in parentheses.

Supplementary Table 3 Comparison of *Z* scores between the NFQ and DMN subnetworks

Participant	NFQ	pDMN	adDMN	vDMN
dAD1	4.15	-2.15	0.61	-2.53
dAD2	-0.47	2.27	2.13	-0.06
dAD3	3.88	-0.53	-0.94	-4.12
dAD4	0.81	-0.45	-1	-1.73
dAD5	5.16	-2.09	-0.44	-1.89
dAD6	3.50	-2.12	-1.48	-1.7
dAD7	1.98	-0.32	-0.92	-2.35
dAD8	1.81	-2.13	-1.02	-2.92
dAD9	1.31	-1.32	-1.05	-1.82
dAD10	-0.07	0.06	-2.11	-0.35
AD1	2.20	-1.92	1.29	-1.55
AD2	3.46	-2.17	-2.43	-1.44
AD3	0.61	0.08	-3.69	0.99
AD4	0.77	1.36	-0.21	-0.55
AD5	2.65	-1.55	-0.28	-1.81
AD6	3.38	-3.36	0.45	-0.23
AD7	1.90	0.46	0.05	-2.48
AD8	-0.91	-1.57	-0.7	-0.77
CU1	1.07	-0.74	1.16	-0.4
CU2	0.53	-0.09	0.54	-1.02
CU3	0.54	0.95	-0.25	-1.15
CU4	-0.53	0.44	0.39	1.5
CU5	-0.57	1.38	-2.17	-0.77

DMN = Default mode network; pDMN = Posterior DMN; adDMN = Anterior dorsal DMN; vDMN = Ventral DMN

Supplementary Table 4 Assessment of site effects in age-adjusted Z scores yielded by quantile regression for all biomarkers of interest

Variable	*F*	*P*	Effect size	Post-hoc
Network failure quotient	0.97	.42	-	-
Cortical thickness region of interest	4.58	.001	0.02	UCLA & WashU > MGH
Left hippocampus volume	1.86	0.12	-	-
Right hippocampus volume	4.94	.001	0.02	UCLA > UMinn
Inferior parietal thickness	2.41	.04		WashU > MGH

*Only cognitively unimpaired controls from Mayo Clinic are included in the comparisons. Mayo Clinic (*N* = 5); Massachusetts General Hospital (MGH; *N* = 162); University of California Los Angeles (UCLA; *N* = 148); University of Minnesota (UMinn; *N* = 205); Washington University (WashU; *N* = 209).

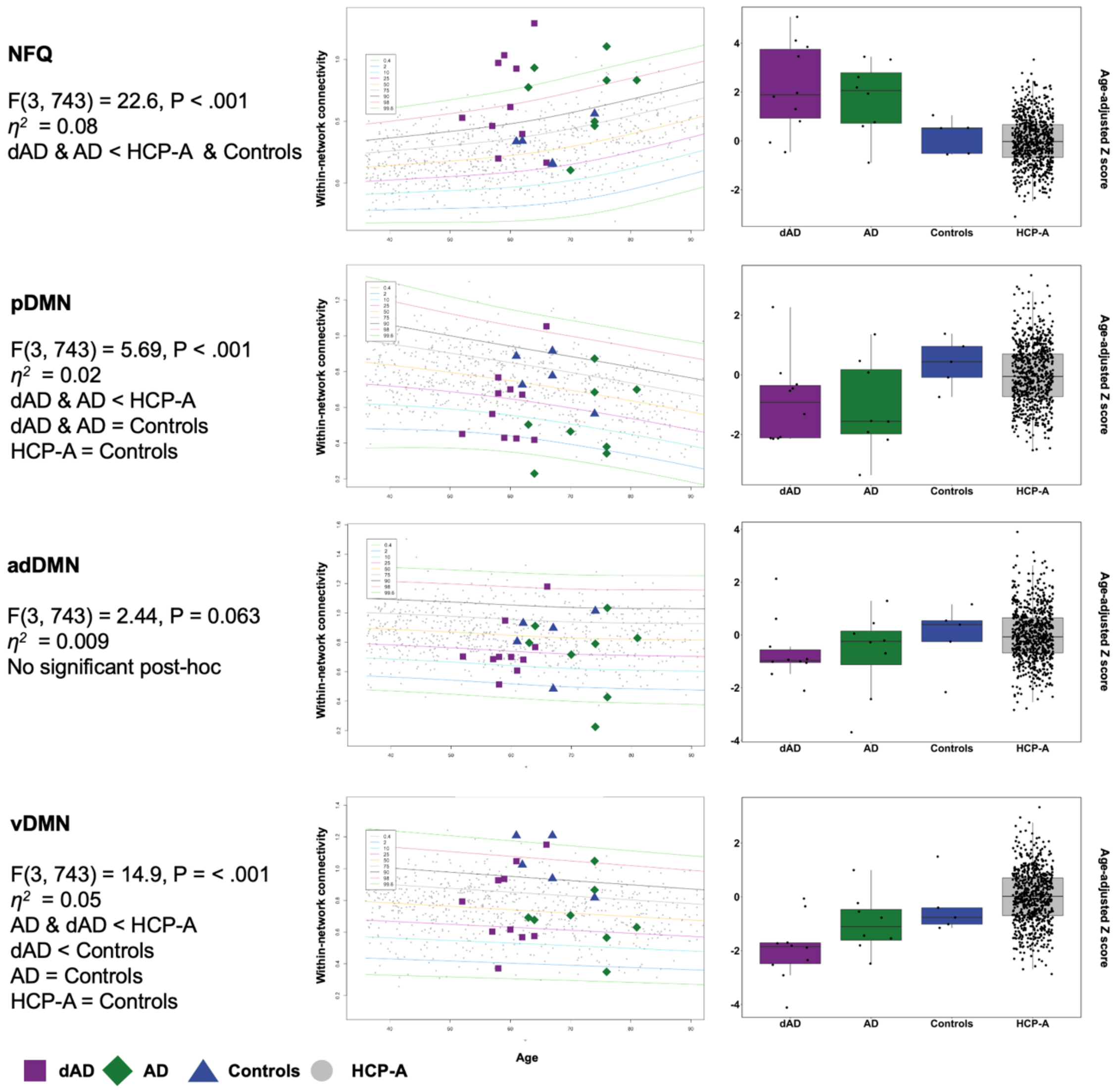

Supplementary Figure 1. Quantile curves and group-wise comparisons on DMN subnetwork within connectivity. Age-adjusted curves are shown for a varying number of centiles in smaller boxes within the plots. dAD = Dysexecutive Alzheimer's disease; AD = Alzheimer's disease; HCP-A = Human Connectome Project-Aging; DMN = Default mode network; pDMN = posterior DMN; adDMN = anterior dorsal DMN; vDMN = ventral DMN.

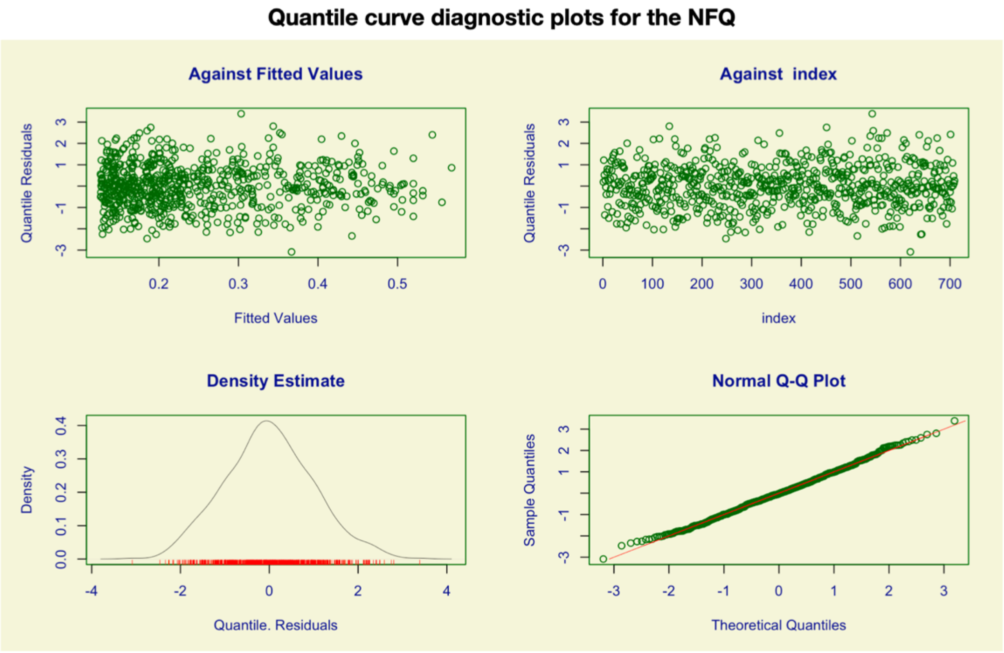

Supplementary Figure 2. Residuals, density and Q-Q plots for the network failure quotient.

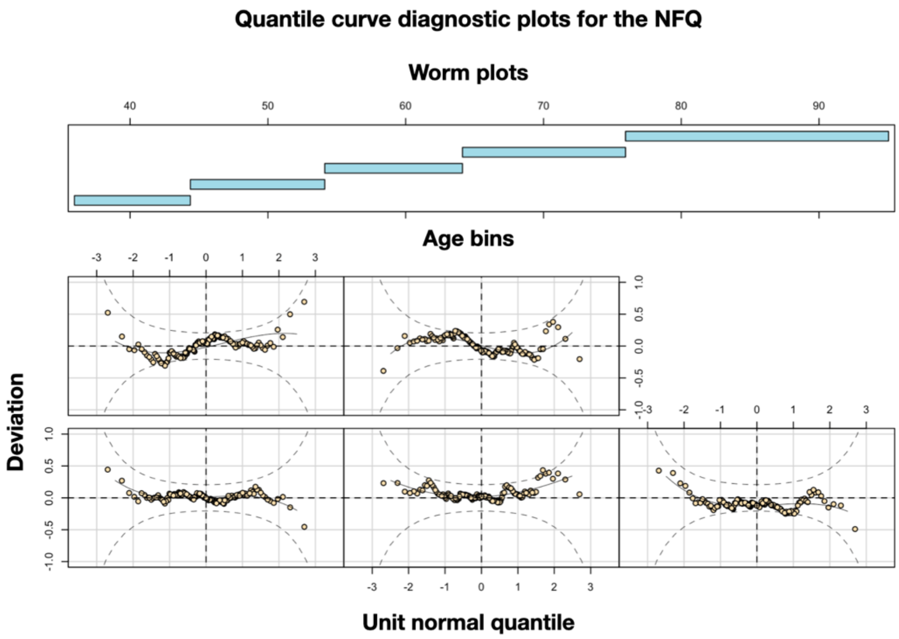

Supplementary Figure 3. Worm plots for the network failure quotient.

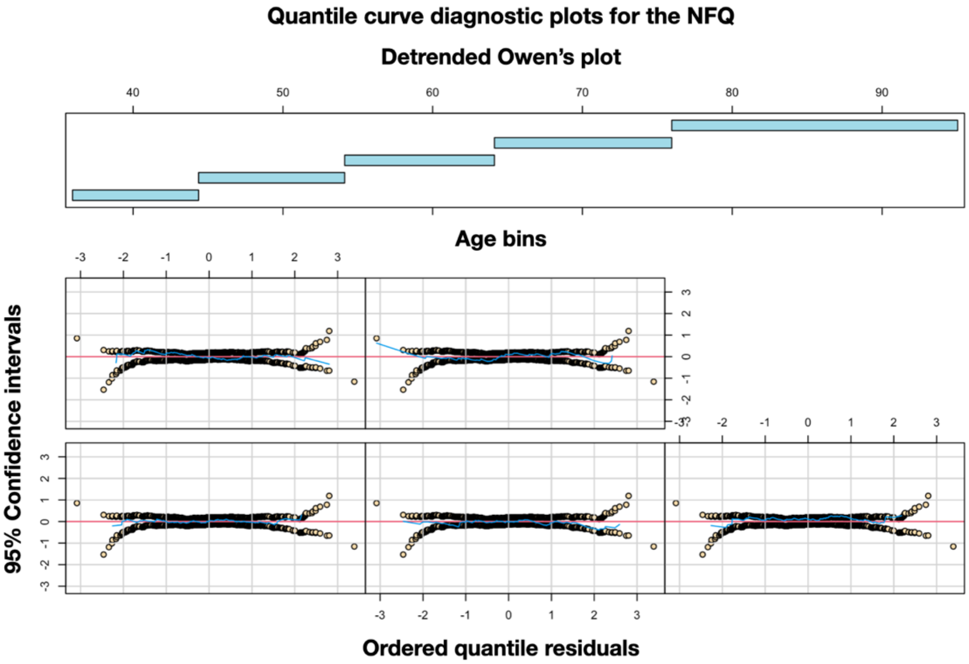

Supplementary Figure 4. Detrended Owen's plot for the network failure quotient.

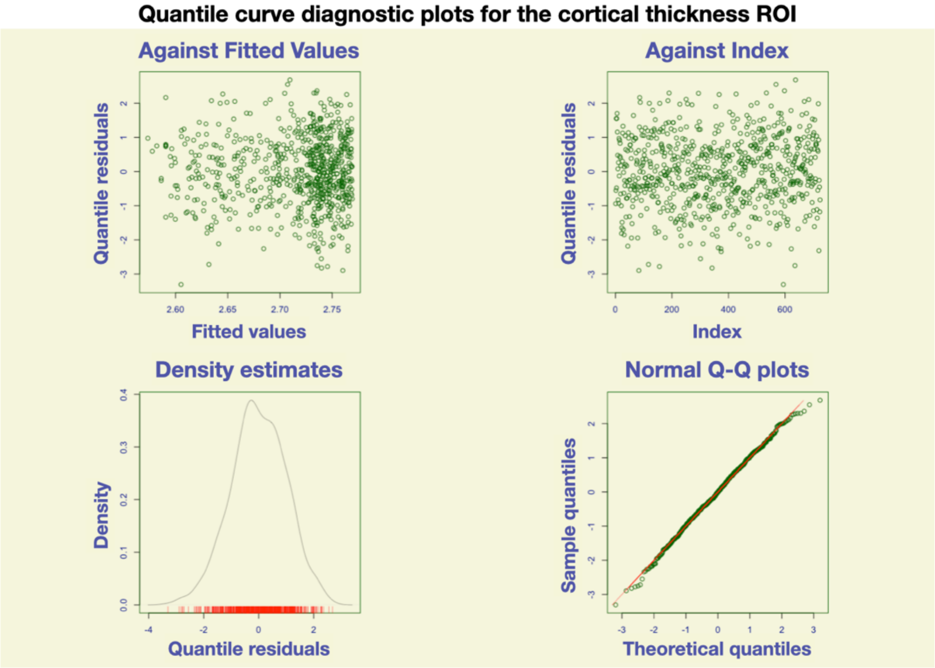

Supplementary Figure 5. Residuals, density and Q-Q plots for the cortical thickness meta-region of interest.

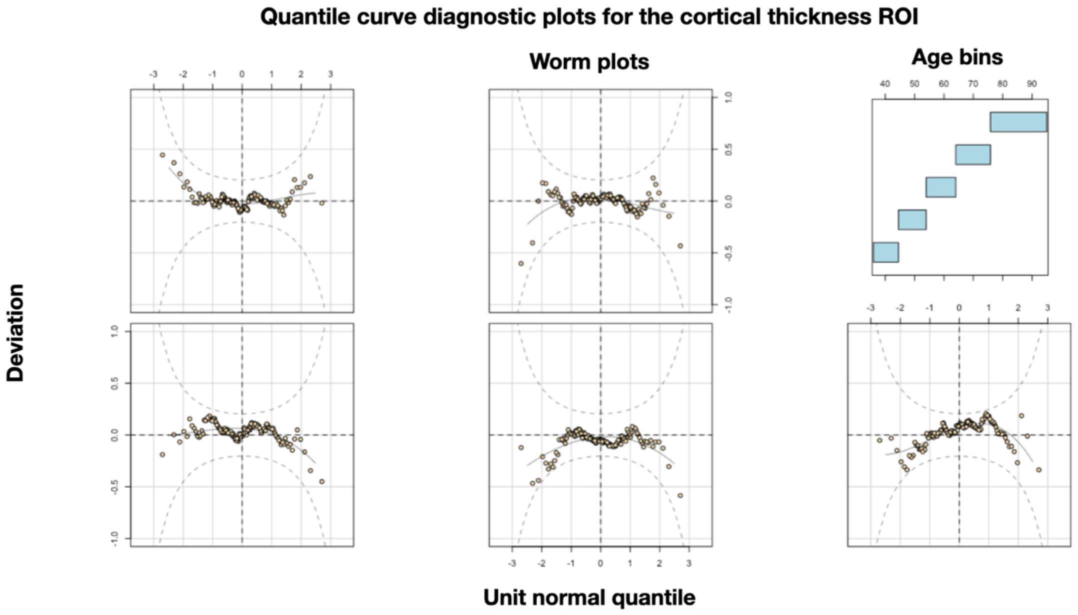

Supplementary Figure 6. Worm plots for the cortical thickness meta-region of interest.

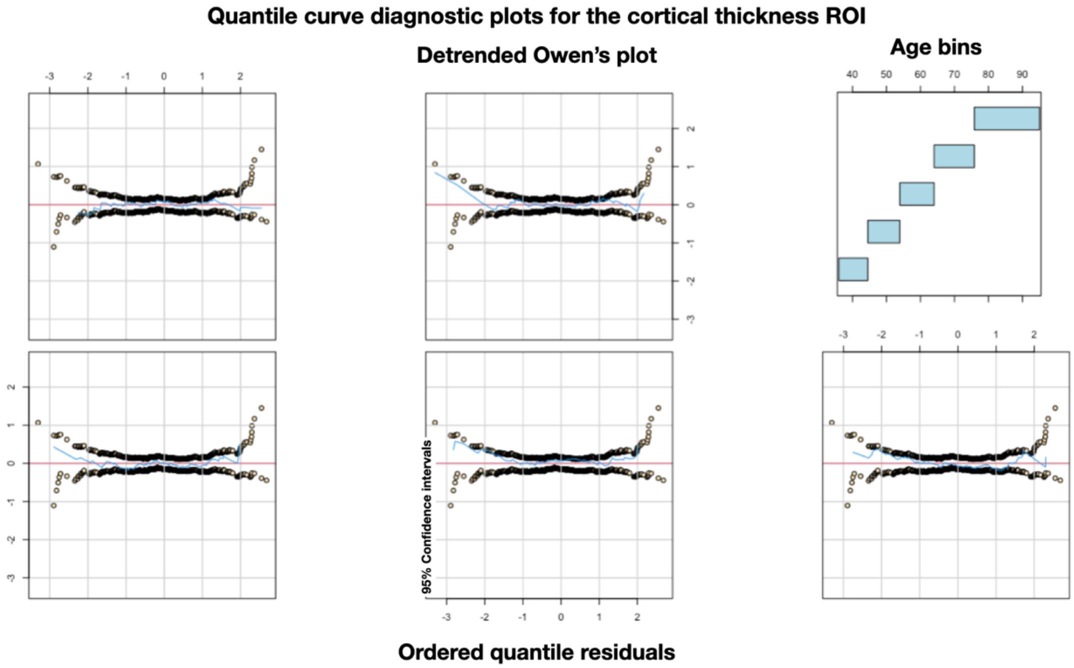

Supplementary Figure 7. Detrended Owen's plot for the cortical thickness meta-region of interest.

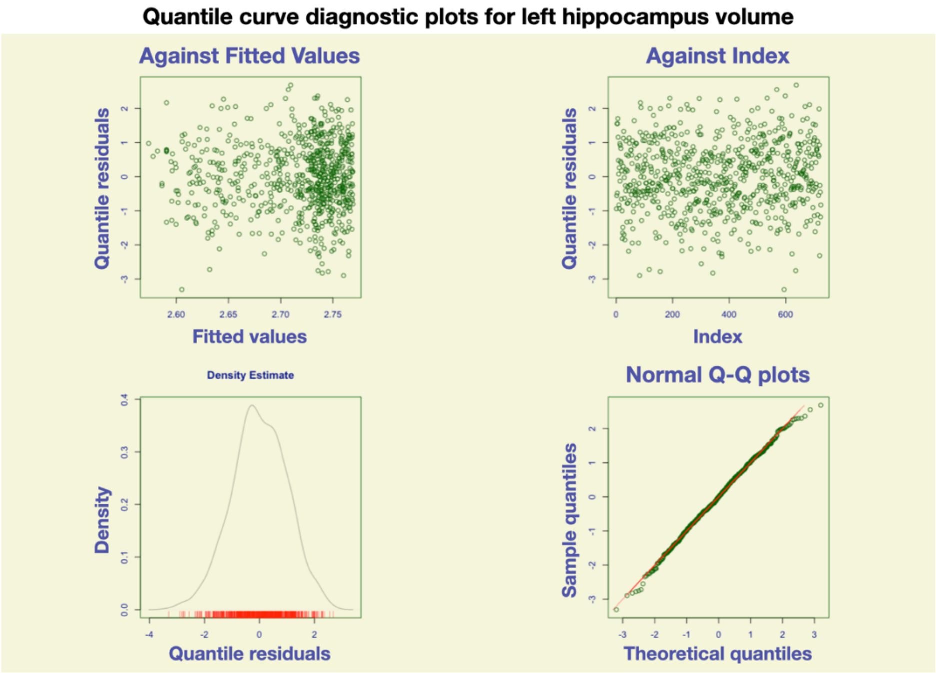

Supplementary Figure 8. Residuals, density and Q-Q plots for the left hippocampal volume.

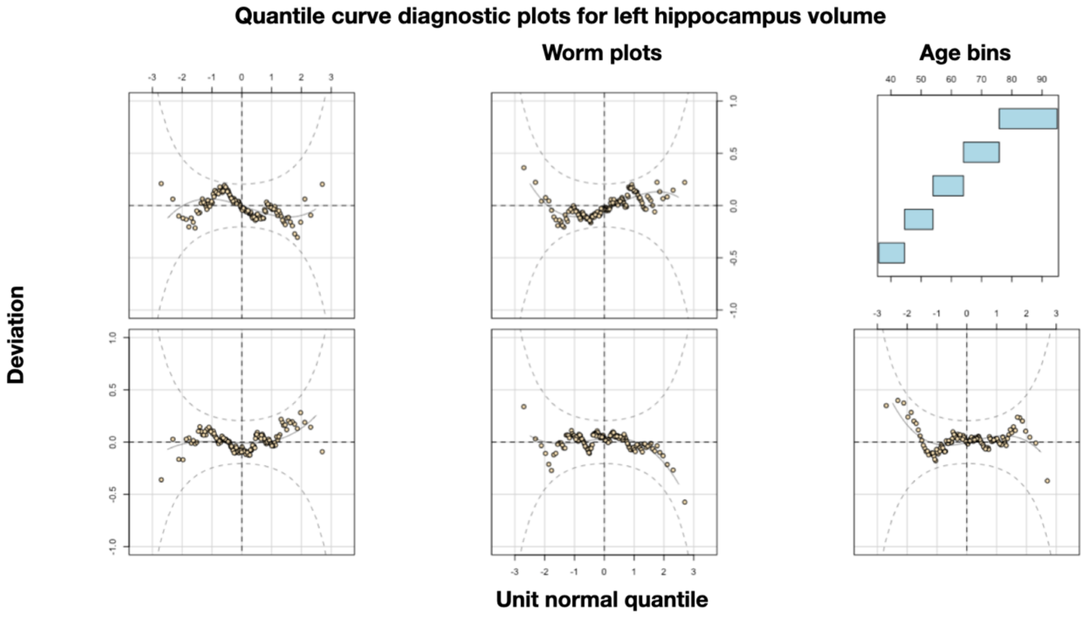

Supplementary Figure 9. Worm plots for the left hippocampal volume.

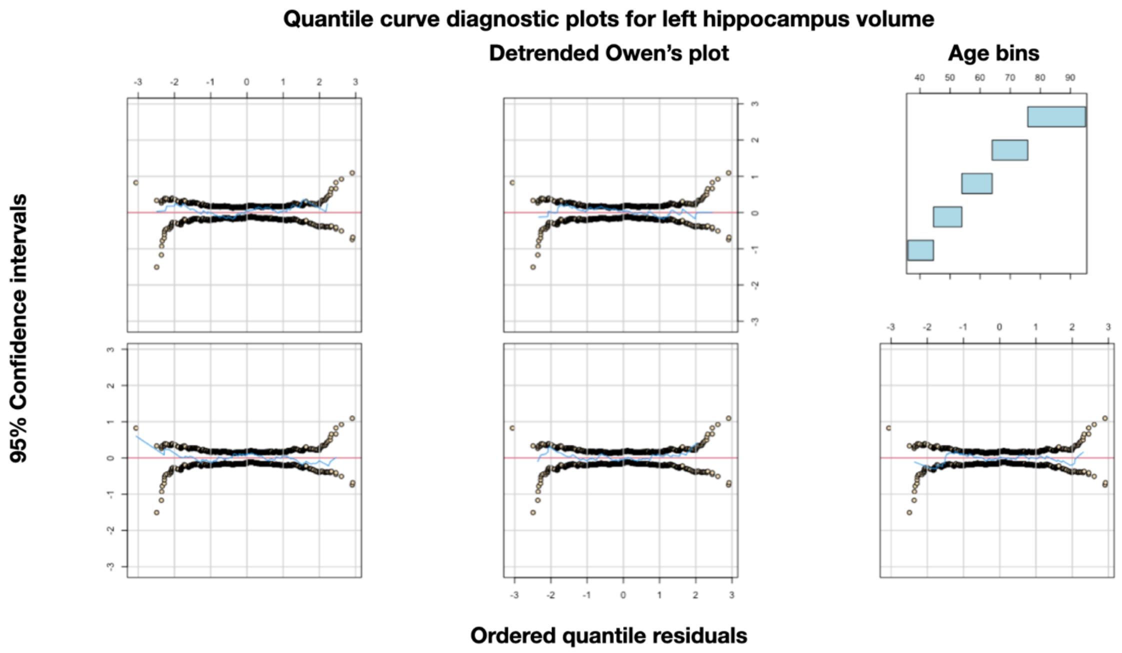

Supplementary Figure 10. Detrended Owen's plot for the left hippocampal volume.

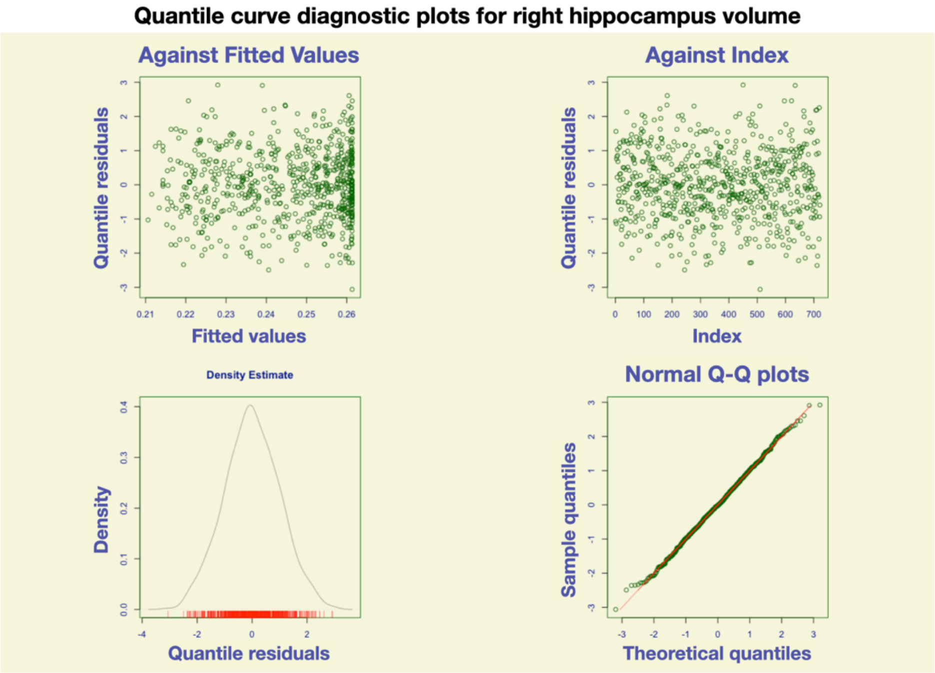

Supplementary Figure 11. Residuals, density and Q-Q plots for the right hippocampal volume.

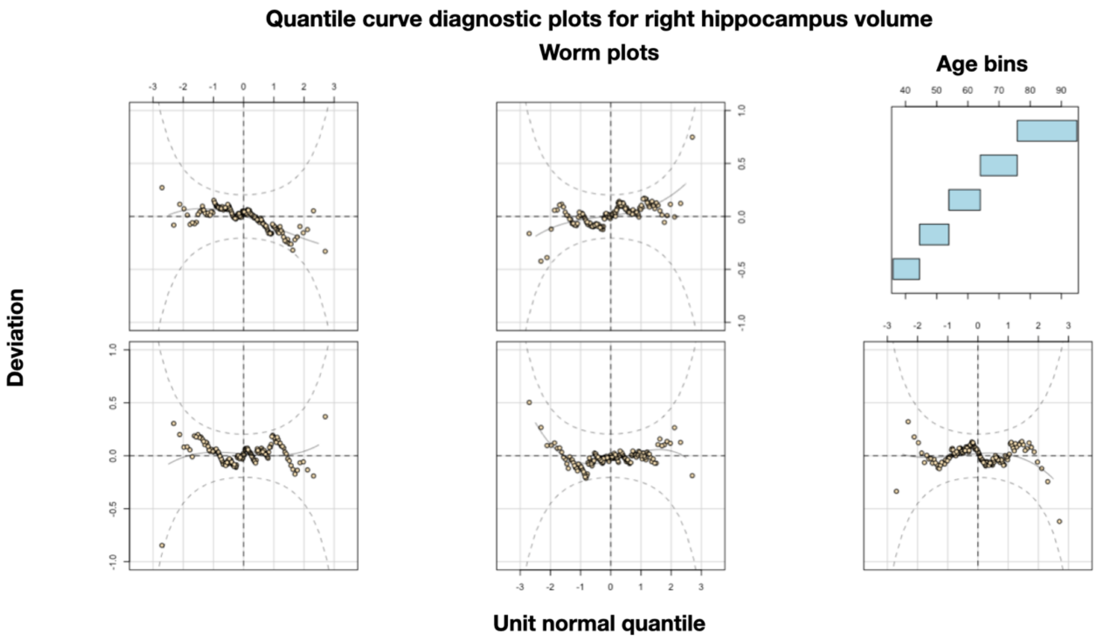

Supplementary Figure 12. Worm plots for the right hippocampal volume.

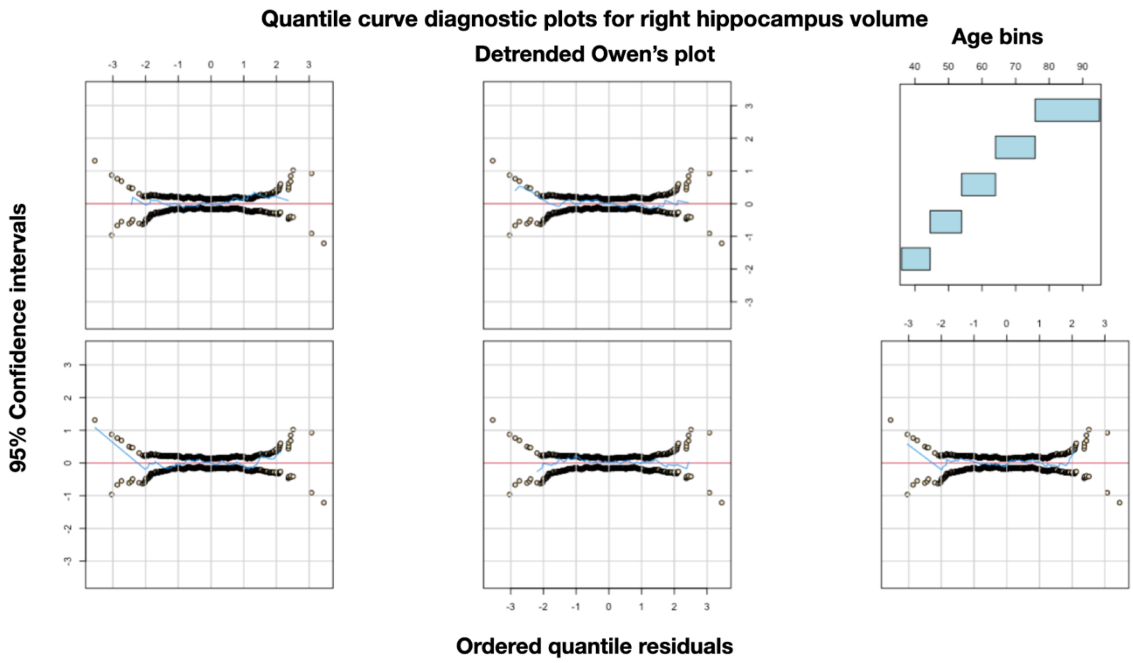

Supplementary Figure 13. Detrended Owen's plot for the right hippocampal volume.

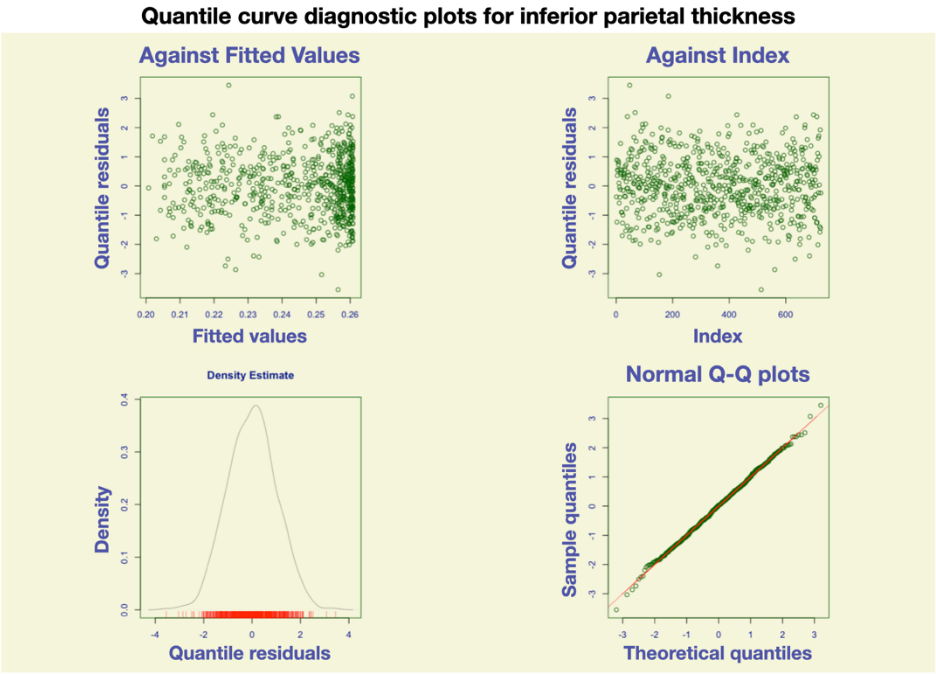

Supplementary Figure 14. Residuals, density and Q-Q plots for the inferior parietal thickness.

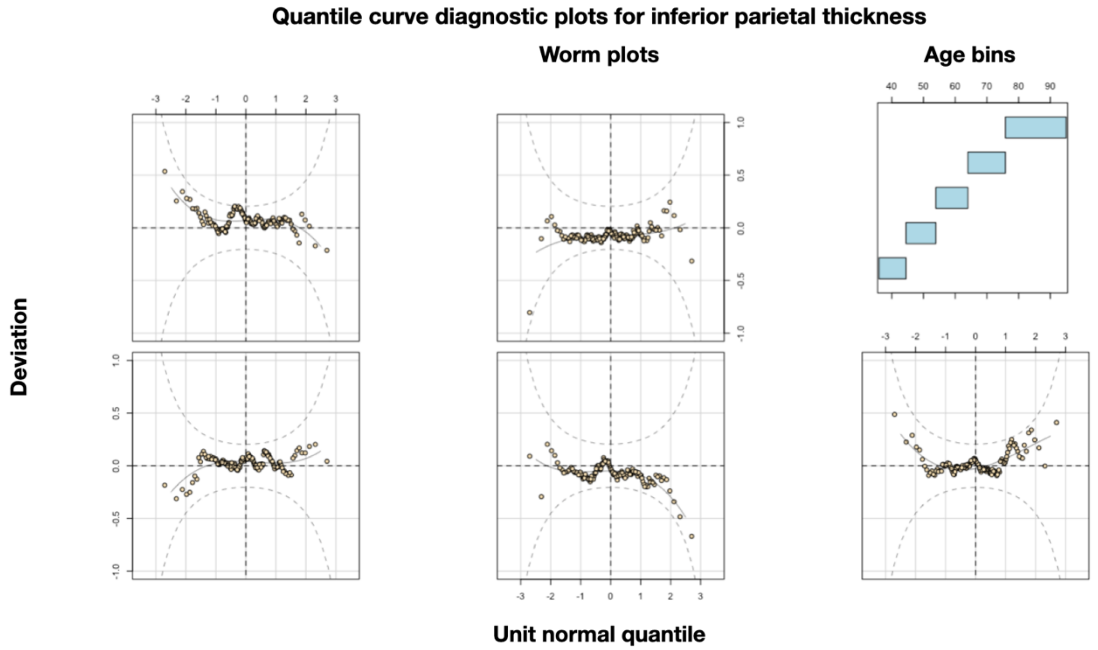

Supplementary Figure 15. Worm plots for the inferior parietal thickness.

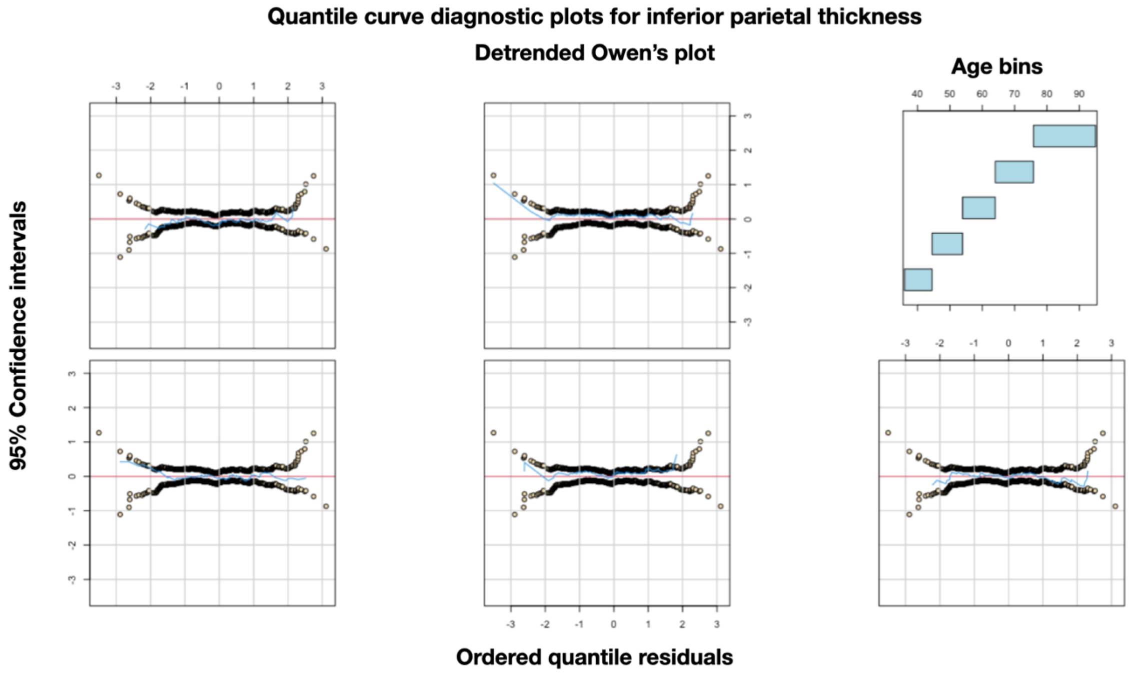

Supplementary Figure 16. Detrended Owen's plot for the inferior parietal thickness.
